# Non-invasive MRI Studies of Ventilatory and Cardiovascular Performance in Edible Crabs *Cancer pagurus* During Warming Under Elevated CO_2_ Levels

**DOI:** 10.3389/fphys.2020.596529

**Published:** 2021-01-11

**Authors:** Bastian Maus, Sebastian Gutsfeld, Christian Bock, Hans-Otto Pörtner

**Affiliations:** ^1^Integrative Ecophysiology, Alfred-Wegener-Institute Helmholtz Centre for Polar and Marine Research, Bremerhaven, Germany; ^2^Department of Biology and Chemistry, University of Bremen, Bremen, Germany

**Keywords:** respirometry, pausing behavior, hypercapnia, non-invasive, crustacea, cardiovascular system, magnetic resonance imaging

## Abstract

The thermal tolerance of marine decapod crustacea is defined through their capacities for oxygen uptake and distribution. High ambient CO_2_ levels were previously shown to reduce hemolymph oxygen levels at enhanced cardiac performance during warming. This study investigated the impacts of warming under two CO_2_ levels on ventilation and hemolymph circulation in edible crabs *Cancer pagurus*. It also highlights changes in the ventilatory and cardiac pauses displayed by Decapoda under routine metabolism. Animals were exposed to step-wise, sub-critical warming (12–20°C over 5 days) under control (470 μatm) and high (1,350 μatm) water *P*CO_2_. Flow-through respirometry was combined with magnetic resonance imaging and infra-red photoplethysmography to allow for simultaneous, non-invasive measurements of metabolic rates ($\dot{M}O_{2}$), ventilation and cardiovascular performance. Crabs spent significantly more time in a low $\dot{M}O_{2}$ state (metabolic pause), when experiencing high CO_2_ conditions above 16°C, compared to normocapnic warming. Heart rates leveled off beyond 18°C at any CO_2_ level. Cardiac output continued to increase with high-CO_2_-warming, due to elevated cardiac stroke volumes. Consequently, temperature-dependent branchial hemolymph flow remained unaffected by CO_2_. Instead, a suppressing effect of CO_2_ on ventilation was found beyond 16°C. These results indicate constrained oxygen uptake at stable cardiovascular performance in a decapod crustacean.

*Cancer pagurus*: urn:lsid:zoobank.org:act:B750F89A-84B5-448B-8D80-EBD724A1C9D4

## Introduction

Edible crabs, *Cancer pagurus*, are abundant in the North Sea, which has been identified as a regional hot spot for global warming (Hobday and Pecl, 2014). Given the severe impacts of marine heatwaves on other commercial crab stocks, it is paramount to improve our understanding of thermal tolerance in economically important species (Chandrapavan et al., 2019). Thermal tolerance in marine crustacea has been linked to hemolymph oxygen levels (*P*_e_O_2_), which are set by the capacities for oxygen uptake, delivery, and demand (Frederich et al., 2000; Frederich and Pörtner, 2000). *P*_e_O_2_ is maximal in the optimum temperature range: for *C. pagurus*, this maximum was found around 10–12°C (Metzger et al., 2007). Thermal limitation sets in when temperature-dependent oxygen demand in the tissues exceeds the oxygen supply *via* the cardiorespiratory system. Under acute warming beyond 16°C, *C. pagurus*’ *P*_e_O_2_ fell (pejus temperature *T*_p_) and at 20°C, it remained at a constant minimum (critical temperature *T*_c_). Generally, warming causes an increase in maximum heart rates and ventilation rates, but beyond *T*_p_, ventilatory and cardiac limitations have been recorded: ventilation failed to increase with temperature in spider crabs *Maja squinado*. Heart rates continue to increase with temperature beyond *T*_p_, but at a much lesser incline than below *T*_p_ (Frederich and Pörtner, 2000).

In crustacea, the heart is the first organ perfused by oxygenated hemolymph from the gills, thus playing a key role in the systemic distribution of oxygen (Taylor, 1982; McMahon and Burnett, 1990). A reduced hemolymph *P*O_2_ measured in the pericardial sinus may be caused by elevated myocardial oxygen consumption or reduced oxygen uptake in the gills. Reduced oxygen uptake may in turn result from low hemolymph flow through the branchial veins or from impaired ventilation, i.e., an impaired ventilation/perfusion ratio (Wheatly and Taylor, 1981). Decapoda are able to independently adjust heart rates and stroke volumes (Airriess and McMahon, 1994; McGaw and McMahon, 1998; Giomi and Pörtner, 2013). Therefore, both parameters need to be measured simultaneously for an accurate assessment of cardiac output and aerobic cardiac performance.

If ambient CO_2_ levels are elevated (hypercapnia), warming causes post-branchial *P*_e_O_2_ to decline at lower temperature thresholds compared to normocapnic warming, shifting the *T*_p_ to lower values and thus narrowing the optimum thermal window (Metzger et al., 2007; Walther et al., 2009). For spider crabs *Hyas araneus*, the reduction in *P*_e_O_2_ under hypercapnia was attributed to elevated heart rates (HR): more frequent contractions of the cardiac muscle may result in a higher oxygen demand of that tissue, reducing the availability of oxygen to the subsequently perfused tissues. The tachycardic effect of hypercapnic warming appears to be dependent on the water CO_2_ level (*P*_w_CO_2_; Walther et al., 2009). While ambient CO_2_ levels of up to 10,000–20,000 μatm did not affect ventilation at control temperatures (Batterton and Cameron, 1978), the combined effects of temperature increases and elevated CO_2_ levels on the interplay of ventilation and branchial perfusion have not been studied to date.

Branchial ventilation and perfusion are functionally linked in Brachyura, so changes in one parameter are paralleled by changes in the other. Regular, synchronous fluctuations in cardiovascular and ventilatory activity were observed in resting Decapoda within their optimum temperature range (McDonald et al., 1977; McMahon and Wilkens, 1977). Periodic fluctuations in whole-animal metabolic rates ($\dot{M}O_{2}$) and HR are displayed by free-moving *C. pagurus* (Ansell, 1973) and also by *Carcinus maenas in situ* (Aagaard, 1996). Over the course of 1 h, peak activities in physiological parameters (like HR) alternate with phases of minimum activities (i.e. bradycardia). This characteristic has thus been termed as pausing behavior (McMahon and Wilkens, 1977) and appears to be reflective of routine metabolism. The periodic changes in ventilatory and cardiac activity may be linked to a pacemaker in the central nervous system, connecting these structures (Taylor, 1982). Energy can be saved during a cardiac and ventilatory pause, since neither hemolymph nor water has to be moved. Consequently, phases of bradycardia and hypoventilation translate into reduced aerobic metabolism, however, pre-branchial hemolymph *P*O_2_ shows peaks and depressions synchronous to the activities of the cardiac and ventilatory systems (Burnett and Bridges, 1981).

Detailed studies exist of the properties and steady-state function of the pausing behavior (McDonald et al., 1980; Burnett and Bridges, 1981; Bradford and Taylor, 1982). However, reports on its response to warming and elevated CO_2_ levels are limited to increased frequencies in phase shifts of pericardial *P*_e_O_2_ as a result of rapid warming (1°C h^−1^; Metzger et al., 2007).

The focus of the present study was to investigate how elevated *P*_w_CO_2_ affects temperature-dependent ventilatory, cardiovascular, and/or aerobic performance in a large crustacean model; these components of the oxygen uptake and delivery system affect thermal tolerance in the edible crab *C. pagurus*. It was hypothesized that exposure to high CO_2_ levels limits the temperature-dependent capacity for performance in one or multiple components of the edible crab’s cardio-respiratory chain. Due to the functional connection of cardiac and ventilatory systems in Decapoda, metabolic rate, ventilatory water flow, and cardiovascular performance were recorded simultaneously in a non-invasive setup. Whole-animal metabolic rates ($\dot{M}O_{2}$) were used as a proxy for aerobic capacity and measured in a flow-through setup. They were recorded in conjunction with cardiovascular and ventilatory performance *via* either magnetic resonance imaging (MRI; for hemolymph flow, ventilatory water flow, and cardiac contractility) or infrared photoplethysmography (IR-PPG; for heart rates, stroke volumes, and cardiac output). Non-invasive measurements of multiple physiological parameters should allow for a closer look at the response of pausing behavior to environmental perturbations. A step-wise temperature increase until maximum summer habitat temperatures was performed under two CO_2_ levels, encountered in the animal’s natural habitat (Artioli et al., 2012; Atamanchuk et al., 2014).

## Materials and Methods

### Experimental Animals

Edible crabs *Cancer pagurus* (Linneaus, 1758) were caught *via* net fishing from R/V Uthörn in July 2017 around the island of Helgoland in the North Sea at depths between 10 and 20 m. They were transported to the aquaria of the Alfred-Wegener-Institute Helmholtz Centre for Polar and Marine Research, Bremerhaven, Germany and kept in natural seawater at 12°C and 32 salinity. Animals were allowed to feed to saturation on *Mytilus edulis* flesh. Food sources were replaced twice a week. To ensure baseline metabolism during measurements, food consumption was stopped 48 h before any experimental treatment by withholding any food from the animals (Ansell, 1973). All applicable international, national, and institutional guidelines for the care and use of animals were followed. All procedures performed involving animals were approved by the ethical standards of the institution at which the studies were conducted. Eight animals (seven females and one male) were used in the experiments, with a mean weight of 330 ± 39 g.

### Experimental Setup and Water Parameters

**Figure 1:**
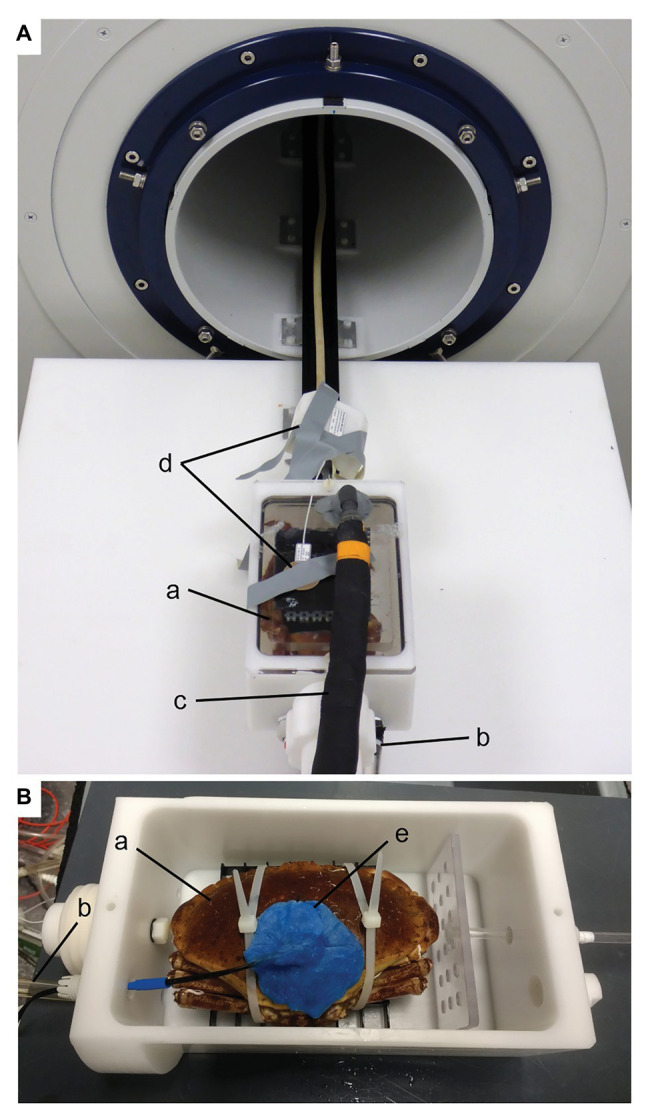
Experimental setup for MRI **(A)** and IR-PPG **(B)** experiments. The animal chamber is placed outside of the magnet in **(A)**. Water is supplied to the crab **(a)** *via* the inlet **(b)** and leaves the chamber *via* a dorsal outlet **(c)**. A surface coil **(d)** for improved signal reception is visible on top of the chamber but was not used for the present experiments. For IR-PPG experiments **(B)**, a larger chamber was used to accommodate the plethysmograph sensor (**e**; see text). Animals were fixed to a plastic grid with cable ties. Once the chamber was closed with a lid similar to the one in **(A)**, it was submerged in seawater and connected to water circulation for respirometry (see Supplementary Figure S2).

Animals were placed in polyurethane chambers to allow for measurements of whole-animal oxygen consumption (metabolic rate, $\dot{M}O_{2}$) with flow-through respirometry (Figure 1). Infra-red photoplethysmography (IR-PPG) was used to determine heart rates (HR), proxies (SVP) for cardiac stroke volume (SV) and cardiac output (CO; Maus et al., 2019a). Cardiac motion, branchial hemolymph flow, and ventilatory water flow were determined non-invasively with the use of MRI (Maus et al., 2019b). Further details on the measurements are described in subsequent sections.

**Figure 2:**
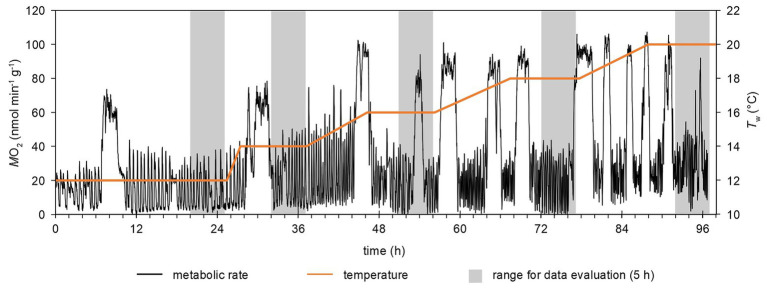
Example of a time-course of temperature changes and metabolic rate. Metabolic rate was continuously recorded in a flow-through setup at a rate of 1 min^−1^. Gray areas highlight the time frames for data evaluation, i.e., the last 5 h of each temperature step. Phases with continuously elevated $\dot{M}O_{2}$ (e.g., around hour 54 at 18°C) were excluded from the analysis (see text).

Temperature control of all natural seawater supply was set to 12°C (Eco 630G and Lauda Gold Series, Lauda-Brinkmann, Delran, NJ, United States). Animals were exposed to this control temperature under either control *P*_w_CO_2_ (400 μatm, normocapnia) or elevated *P*_w_CO_2_ (1,350 μatm, hypercapnia) for 1 day. All seawater reservoirs were covered with a lid to minimize CO_2_ exchange between atmosphere and water. At the defined *P*_w_CO_2_, water temperatures were increased at a rate between 0.17 and 1°C h^−1^. The rate of temperature change allowed for 10 h plateau phases at 14, 16, 18, and 20°C during working hours (Figure 2). After 10 h at 20°C, water temperature (*T*_w_) was lowered to the control temperature of 12°C within 8 h (1°C h^−1^). One experimental run lasted 5 days. Three animals were subjected to IR-PPG experiments and five animals to MRI experiments. All techniques applied here were non-invasive, so normocapnic and hypercapnic experiments in each setup could be replicated on the same individuals, separated by at least 1 week of recovery in the animals’ holding aquaria (Field, 1977). Two females in the MRI experiments were only exposed to either normocapnic or hypercapnic warming.

Water *P*CO_2_ was adjusted with an air-CO_2_ mix, set by a gas mixing device (PR4000; MKS, Andover, MA, United States). The water carbonate chemistry was monitored with a combined CO_2_ probe (CARBOCAP GMP343, Vaisala, Helsinki, Finland) and CO_2_ meter (CARBOCAP GM70, Vaisala), together with water pH measurements. The pH meter (pH3310, WTW, Weilheim, Germany) was calibrated in NIST buffers at 12 and 20°C to measure seawater pH (pH_w_) at these temperatures. pH_w_ was converted to the free scale, with corrections for ionic strength and a reference pH in artificial seawater (Dickson, 2010; Waters and Millero, 2013). Water [HCO_3_^−^], total alkalinity (TA) and total dissolved inorganic carbon (DIC) were calculated in mmol per kg seawater with CO2Sys (Pierrot et al., 2006) with *K*_1_ and *K*_2_ from Millero (2010), *K*SO_4_ from Dickson (1990) and [B]_T_ from Uppström (1974).

### Respirometry

Whole-animal oxygen consumption was determined in a flow-through setup (Steffensen, 1989), incorporated into both, the MRI-experiments (similar to Mark et al., 2002) and IR-PPG experiments (similar to Zittier et al., 2015). Oxygen saturation (*P*_w_O_2_ in percent air saturation, corrected for atmospheric and water vapor pressure) was measured with temperature-compensated fiber-optical oxygen sensors (FIBOX 3; PreSens, Regensburg, Germany) directly before and after the animal chamber at a frequency of 1 min^−1^, using software PSt3 (v7.01, PreSens). Sensors were calibrated at 12°C for 0% O_2_ saturation in N_2_-gas and 100% saturation in the aerated flow-through system before each animal experiment. To reduce measurement errors during warming steps, another calibration for 100% saturation was conducted at 16°C. With the exception of this calibration step, *P*_w_O_2_ was measured continuously for 5 days. From the difference in oxygen partial pressure ∆*P*_w_O_2_ between inflow and outflow from the chamber, $\dot{M}O_{2}$ was calculated as

(1) $$\dot{M}O_{2}=\frac{△P_{w}O_{2}•\alpha_{O_{2}}•FR}{w_{f}}$$

where *FR* is the rate of water flow through the chamber (ml min^−1^) and *w*_f_ is animal fresh weight. Temperature-dependent oxygen solubility α_O2_ (μM torr^−1^) was taken from Boutilier et al. (1984). $\dot{M}O_{2}$ data were corrected for delays in water mixing in both experimental setups, determined in a separate experiment with hypoxic seawater at different water flow speeds. These time-corrected values for $\dot{M}O_{2}$ were aligned with the MRI or IR-PPG data, allowing for a temporal correlation between whole-animal oxygen consumption and cardiovascular performance. Previous tests confirmed a 3–4 min delay of the *P*_w_O_2_ signal between inlet and outlet optode, requiring a shift of $\dot{M}O_{2}$ data by this time frame, relative to immediate cardiovascular measurements. Whole-animal oxygen consumption integrates all aerobic processes in an animal and was therefore used to compare the physiological states of the animals in the different measurement setups (see below). In accordance with literature reports, phases of periodic $\dot{M}O_{2}$ fluctuations were deemed routine activity (McDonald et al., 1980) and shall be the foundation for the subsequent analysis.

### Magnetic Resonance Imaging

*In vivo* MRI experiments were conducted in a 9.4 T horizontal animal scanner with a 30 cm bore (BioSpec 94/30 Avance III, Bruker BioSpin, Rheinstetten, Germany, RRID:SCR_018054). MRI allows for repeated, non-invasive measurements of the performance of the crustacean cardiovascular system (Maus et al., 2019b). Briefly, individuals were placed inside the MRI scanner in a respiration chamber (Figure 1A, polyurethane; *V*_chamber_ = 1 L, including Tygon© tubing between oxygen optodes, see above) and continuously supplied with seawater from an aerated 40 L header tank, controlled for *P*_w_CO_2_ and temperature. Water passed through the chamber at a rate of 200–300 ml min^−1^. It was collected in an overflow basin and then pumped back into the header tank *via* a peristaltic pump. To keep the animals’ carapace in place, it was attached to the removable lid with Velcro©. Three animals were subjected to MRI experiments under both hypercapnic and normocapnic conditions. Two other crabs were only subjected here to either normocapnic or hypercapnic warming (*n* = 4 per CO_2_ treatment).

Magnetic resonance images were acquired with a 154-mm-diameter ^1^H-tunable transmit-receive volume resonator, pre-configured for application on seawater samples (Bruker BioSpin). Adjustments of the magnetic field homogeneity, receiver gain, and reference power followed standardized protocols, integrated into the software ParaVision (v6.0.1; Bruker BioSpin).

Sagittal and coronal *T*_1_-weighted overview scans revealed the location of the heart and major blood vessels in the animal, such as the *arteria sternalis* and branchial veins. The scan parameters were; sequence = flow-compensated fast low-angle shot (FcFLASH); echo time (TE) = 12 ms; repetition time (TR) = 103.4 ms; flip angle = 60°; slice thickness (SI) = 1.5 mm; field of view (FOV) = 120 × 60 mm^2^; 256 × 128 pixels; and eight averages. Visual inspection of these overview scans confirmed developed gonads and no apparent differences in size and structure of the cardiac muscle in all experimental animals.

Ventilatory water flow and hemolymph flow velocities in the branchial veins and *arteria sternalis* were quantified using flow-weighted gradient-echo MRI (Bock et al., 2001; Maus et al., 2019b; Supplementary Figure S1). Scan parameters: phase-contrast MRI (FLOWMAP); TE = 12 ms; TR = 25 ms; flip angle = 30°; SI = 1.2 mm; FOV = 100 × 100 mm^2^; 256 × 256 pixels; 16 averages; and velocity encoding = 12 cm s^−1^. Hemolymph and water flow velocities were determined in manually set regions of interest (ROI) in the Image Display and Processing platform within ParaVision. Hemolymph flow was quantified in the inner (efferent) branchial veins of gills 6–8 on both sides of the animals, as well as the sternal artery. Left and right branchial hemolymph flow velocities were averaged for these three gills, respectively. Ventilatory water flow was taken from the anterior transition of the branchial chamber to the excurrent channel. Water flow through the branchial chamber is linearly correlated with scaphognathite beating during forward ventilation (McDonald et al., 1980). The slice position was adjusted to cover a coronal cross-section of the respective blood vessels and the excurrent channel. Since the side of perfusion/ventilation was arbitrary and subject to change during the acquisition, the flow velocities for left and right gill chambers are presented here as sums for both sides.

Axial single-slice IntraGate© CINE MRI scans were performed to visualize cardiac contractility directly. Contractility is defined here as change in distance between the two lateral ostia between end-diastolic and end-systolic phases. The scan parameters were: sequence = IgFLASH; TE = 3.885 ms; TR = 8 ms; flip angle = 60°; SI = 1.2 mm; FOV = 120 × 60 mm^2^; 256 × 125 pixels; 100 oversampling steps; and 10 cardiac frames. Contractility was determined through the difference in lateral diameter of the ventricle between the two in-plane ostia between end-diastole and end-systole using ParaVision’s image viewer.

Magnetic resonance images were recorded for the last 4–5 h of each temperature step (i.e., 12, 14, 16, 18, and 20°C; Figure 2). One CINE scan every 15 min (four per hour) was followed by three flow velocity measurements (12 per hour).

### Infrared Photoplethysmography

Heart rates (HR) were derived together with a stroke volume proxy (SVP) from IR-PPG recordings of individual crabs (isiTEC, Bremerhaven, Germany; Depledge, 1984). The animal chamber for these measurements was slightly larger than the one used for MRI experiments (*V* = 1.87 L) and submerged in a 40 L seawater tank (Figure 1B; Supplementary Figure S2). Aeration and control of *T*_w_ and *P*_w_CO_2_ was achieved as described above. The wire of the IR-PPG sensor was guided out of the chamber through a water-tight hole. Otherwise, the chamber had one inlet and one outlet to allow for the flow-through respirometry setup. Animal movement in the chamber was restricted by strapping the animal to a plastic grid with cable ties (Frederich and Pörtner, 2000). The IR-PPG sensor was attached to the cardiac region of the carapace using superglue and dental wax (Figure 1B). Signals were amplified with a 5 V amplifier, digitized by an A/D converter (Powerlab/8SP; ADInstruments, Dunedin, New Zealand) and recorded in LabChart (v7, ADInstruments, RRID:SCR_017551) at a rate of 1 k s^−1^. Similar to the MRI experiments, only the last 5 h were analyzed per temperature step.

Raw IR-PPG signals were smoothed (0.1 s) and filtered for noise (0.02 V). Positive peaks representing heart contraction were defined at a minimum peak height of 1.5–3.4-fold standard deviation and counted at 1-min-intervals. Stroke Volume Proxies (SVP) were determined from the signal integral of the raw signal relative to the absolute minimum of a 1-min-interval using the mid-point approach (Maus et al., 2019a). SVP were normalized to a mean SV of 0.2 ml beat^−1^ (Burnett et al., 1981; Bradford and Taylor, 1982) at 12°C normocapnia and hypercapnia to account for different signal quality. This showed relative SV changes to 12°C under both CO_2_ conditions and allowed for the calculation of cardiac output (CO) as the product of HR and SV.

### Statistics and Data Analysis

For each combination of animal, *T*_w_ and *P*_w_CO_2_, the total range of $\dot{M}O_{2}$ was divided into quarters. The time spent in the highest and lowest 25% of the total range is given as a percentage of total time for each combination of *T*_w_ and *P*_w_CO_2_. This allowed for the analysis of the pattern of the changes over time (i.e., time spent at maximum or minimum activity of the periodic changes). Temperature-dependent differences in these grouped times at one *P*_w_CO_2_ were compared with a Kruskal Wallis test (ANOVA-on-ranks) and a Tamhane T2 *post hoc* test. CO_2_-dependent differences at one temperature were tested with the Wilcoxon rank-sum-test.

The distribution of data points for any combination of *T*_w_ and *P*_w_CO_2_ was tested for normal distribution (Shapiro-Wilk test) and equal variance (Levene’s test). Changes in water carbonate chemistry and cardiac lateral contractility were investigated over the temperature increase at set *P*_w_CO_2_ by comparing values for 12 and 20°C and for differences between normocapnic and hypercapnic conditions at set temperatures (Student’s *t*-test).

The inherent variability over time in metabolic rate, ventilation, hemolymph flow and cardiovascular activity led to non-normal distribution of values even at control conditions. The effects of temperature and hypercapnia on these parameters were investigated with a linear mixed effects model (R Project, RRID:SCR_001905; package lme4; Bates et al., 2015). Prior to calculations, the data were standardized. Temperature, CO_2_ level (i.e., normocapnia or hypercapnia) and their interaction were treated as fixed effects. Animals were treated as random effects with random intercepts. This way, for each physiological parameter, the repeated measurements on one individual and inter-individual baseline activities could be accounted for. Accordingly, collective analyzes of physiological responses to *T*_w_ and *P*_w_CO_2_ were possible. The inclusion of random slopes was omitted to avoid overfitting of the model, which leads to a significant loss in statistical power (inflated Type II error rate; Matuschek et al., 2017). Given the large amount of observations, the assumptions for homoscedasticity and normality of the standardized residuals are fulfilled sufficiently (see Supplementary Figure S3) and statistically relevant results with low Type I error rates can be retrieved with correct covariance structures despite small animal numbers (Oberfeld and Franke, 2013). Suitability of the model with the chosen effects was verified *via* likelihood-ratio-tests against models without these effects (for detailed results, see Supplementary Table S1). The model code and detailed model estimates can be retrieved from the supplementary material (estimates; Supplementary Table S2). Model estimates were extracted using the R package stargazer (Hlavac, 2018). Differences between combinations of temperature and normocapnic/hypercapnic treatments were determined through an all-pairwise-comparison of means with Tukey’s HSD correction using the R package emmeans (Lenth et al., 2020; Supplementary Table S3).

Physiological parameters are visualized through violin plots, showing the probability density distribution of the recorded data. The width of a plot corresponds to the number of measurements at this value, where narrower violin width indicates fewer instances or data points and wider width shows a larger number of instances. Since data for a specific combination of *T*_w_ and *P*_w_CO_2_ were recorded from a time series, a wider violin can also be interpreted as more time spent at this value (resulting in more frequent measurements).

The linear correlation between ventilatory water flow and branchial hemolymph flow was assessed from phase-contrast MRI using Pearson’s correlation coefficient. Except for the linear mixed effects model, all statistical analyzes were performed using SPSS (v25, IBM Corp., Armonk, NY, USA, RRID:SCR_002865). The level of significance for all tests was *α* = 0.05. If not stated otherwise, values are given as means ± standard deviation.

## Results

### Seawater Parameters

The seawater carbonate chemistry under different CO_2_ treatments is presented in Table 1 for 12 and 20°C. Significant differences in *P*_w_CO_2_ and seawater pH (pH_w_; free scale) were found between the two CO_2_ treatments at both temperatures. Total alkalinity (TA) and dissolved inorganic carbon (DIC) were the most variable components of the carbonate system between all groups, but changes were within 400 μmol kg^−1^ (<20%) throughout. At a given *P*_w_CO_2_, carbonate chemistry remained generally unchanged between 12 and 20°C. With one CO_2_ treatment, the significant differences in pH_w_ and *P*_w_CO_2_ between 12 and 20°C were small on absolute scales, especially compared to the differences between the two different CO_2_ levels. Despite nominally identical conditions, high-CO_2_-conditions resulted in pH_w_, [HCO_3_^−^]_w_, TA, and DIC values were significantly different between MRI and IR-PPG experiments; still, absolute differences were not larger than between temperature treatments and thus also considered negligible. Random samples at intermittent temperatures (14, 16, or 18°C) have confirmed stable seawater conditions per treatment during the different warming steps in both experimental series (data not shown).

**Table 1:** Seawater parameters during MRI and IR-PPG experiments.

setup	Condition	*T*_w_ (°C)	*S*	*P*_w_CO_2_ (μatm)	pH_w_ (free scale)	[HCO_3_^−^]_w_ (mmol kg^−1^)	TA (mmol kg^−1^)	DIC (mmol kg^−1^)
MRI	Normocapnia	11.85 ± 0.19	32.75 ± 0.25	478 ± 27	8.06 ± 0.04	2.43 ± 0.10	2.86 ± 0.15	2.63 ± 0.12
		19.72 ± 0.29^a^	33.00 ± 0.59	485 ± 18	8.08 ± 0.04	2.40 ± 0.11	2.98 ± 0.18	2.67 ± 0.14
	Hypercapnia	11.50 ± 0.41	32.92 ± 0.91	1,379 ± 53^*^	7.61 ± 0.01^*^	2.47 ± 0.10	2.62 ± 0.10^*^	2.59 ± 0.10
		19.74 ± 0.35^a^	33.02 ± 0.86	1,364 ± 51^†^	7.66 ± 0.02^†,a^	2.59 ± 0.12	2.83 ± 0.14^a^	2.73 ± 0.13
IR-PPG	Normocapnia	12.07 ± 0.15	33.5 ± 0.1^b^	483 ± 21	8.10 ± 0.02	2.23 ± 0.18	2.61 ± 0.22	2.41 ± 0.20
		20.03 ± 0.06^a^	33.57 ± 0.12	458 ± 33	8.17 ± 0.03^a,b^	2.26 ± 0.13	2.83 ± 0.18	2.52 ± 0.16
	Hypercapnia	12.07 ± 0.21^b^	33.7 ± 0.2	1,375 ± 13^*^	7.64 ± 0.19^*^	2.21 ± 0.10^b^	2.34 ± 0.11^b^	2.32 ± 0.10^b^
		19.97 ± 0.15^a^	33.78 ± 0.14	1,324 ± 14^†a^	7.714 ± 0.03^†,a,b^	2.28 ± 0.14^b^	2.48 ± 0.16^b^	2.40 ± 0.15^b^

Values are means ± standard deviation for 3–5 experimental runs. Water parameters were determined at 12°C (start of temperature ramp) and 20°C (end of temperature ramp). Individual measurements at intermediate temperature steps confirmed stable values for each treatment. [HCO_3_^−^]_w_, TA and DIC are given as mmol per kg seawater. All tests were *t*-tests at *α* = 0.05.

* Significant differences between hypercapnic conditions and normocapnic conditions at 12°C.

† Significant differences between hypercapnic conditions and normocapnic conditions at 20°C.

a Significant differences between 12 and 20°C at set *P*_w_CO_2_.

b Significant differences between MRI and IR-PPG experiments at given T_w_ and *P*_w_CO_2_.

### Time Course Variations of Metabolic Rate, Ventilation, and Cardiac Performance

**Figure 3:**
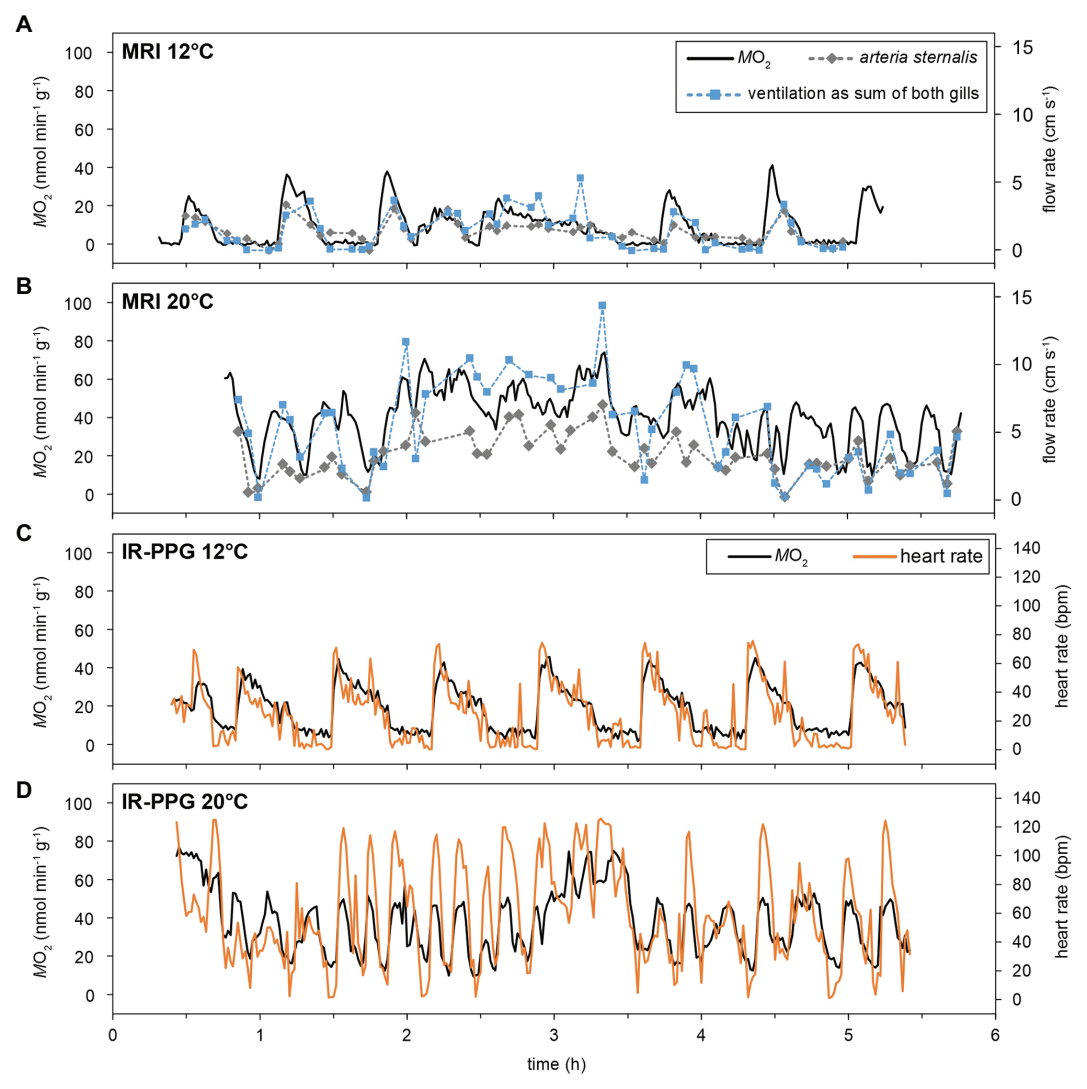
Effect of temperature on the time-course of oxygen consumption, ventilation, and cardiovascular performance. MRI experiments allowed for simultaneous recording of $\dot{M}O_{2}$, ventilatory water flow (as sum of both gills), as well as hemolymph flow in selected vessels (**A,B**; for vessels please check Supplementary Figure S1). To improve visibility, only the flow in the *arteria sternalis* is depicted, but its time course is matched by branchial hemolymph flow. IR-PPG experiments determined $\dot{M}O_{2}$ together with heart rates **(C,D)**. Metabolic rates and heart rates could be recorded at a rate of 1 min^−1^, whereas marks for flow velocities indicate the endpoints of a 3-min measurement. The figure shows recordings obtained from two different individuals for the two setups, however, recordings for 12 and 20°C are from the same animal within one setup.

Over the entire temperature range, resting animals showed periodic variations in the metabolic rate ($\dot{M}O_{2}$), with values fluctuating between short, pronounced maxima, and values close to 0 nmol min^−1^ g^−1^ (at least for the lower temperatures, Figure 2). The amplitude of these periodic changes increased during warming, mainly driven by rising maxima. Minimum $\dot{M}O_{2}$ levels close to 0 nmol min^−1^ g^−1^ were not found above 18°C under normocapnia. On average, 1–3 peak oxygen consumption pulses per hour were found at 12°C, irrespective of *P*_w_CO_2_. The shape of $\dot{M}O_{2}$ changes was similar in the MRI and the IR-PPG setups, and across animals (Figure 3). At 12°C, maxima had a short duration (<10 min), typically followed by a steady decline to baseline levels and a phase of nearly undetectable oxygen consumption (~10 min each). The transition back to maximum values was much faster than the subsequent decline (Figures 3A,C). The time course of changes in $\dot{M}O_{2}$ was mirrored by changes in ventilation activity, hemolymph flow, and heart rate (HR, Figure 3). Most likely due to the lower temporal resolution of the flow measurement technique, the changes in ventilation and hemolymph flow did not perfectly match the pattern in $\dot{M}O_{2}$. Still, periods of low $\dot{M}O_{2}$ were defined by severe hypoventilation and bradycardia. Unilateral ventilation and perfusion of branchial veins were usually found during these phases of fluctuating activities.

With rising temperatures, the fluctuating pattern in activity remained persistent but periodic changes were less clearly defined than at 12°C. $\dot{M}O_{2}$ displayed fluctuations even at 20°C in both setups and its changes were still mirrored by fluctuating ventilation, hemolymph flow, and heart rate (Figures 3B,D). The frequency of activity changes increased with rising temperatures, but in different animals, the periodicity was lost to various degrees. Warming also resulted in more frequent phases of spontaneously elevated activity (Figures 2, 3B,C) when whole-animal activity (i.e., metabolic, ventilatory, circulatory, and cardiac performance) remained high for several hours. Bilateral ventilation and branchial perfusion were typical for these phases. Since phases of spontaneously elevated activity occurred randomly and had different magnitudes and durations, they were excluded from the subsequent analyzes (as an example see hours 2–4 in Figure 3B), focusing instead on routine activity levels across treatments.

### Metabolic Rate Under Warming and Elevated CO_2_

**Figure 4:**
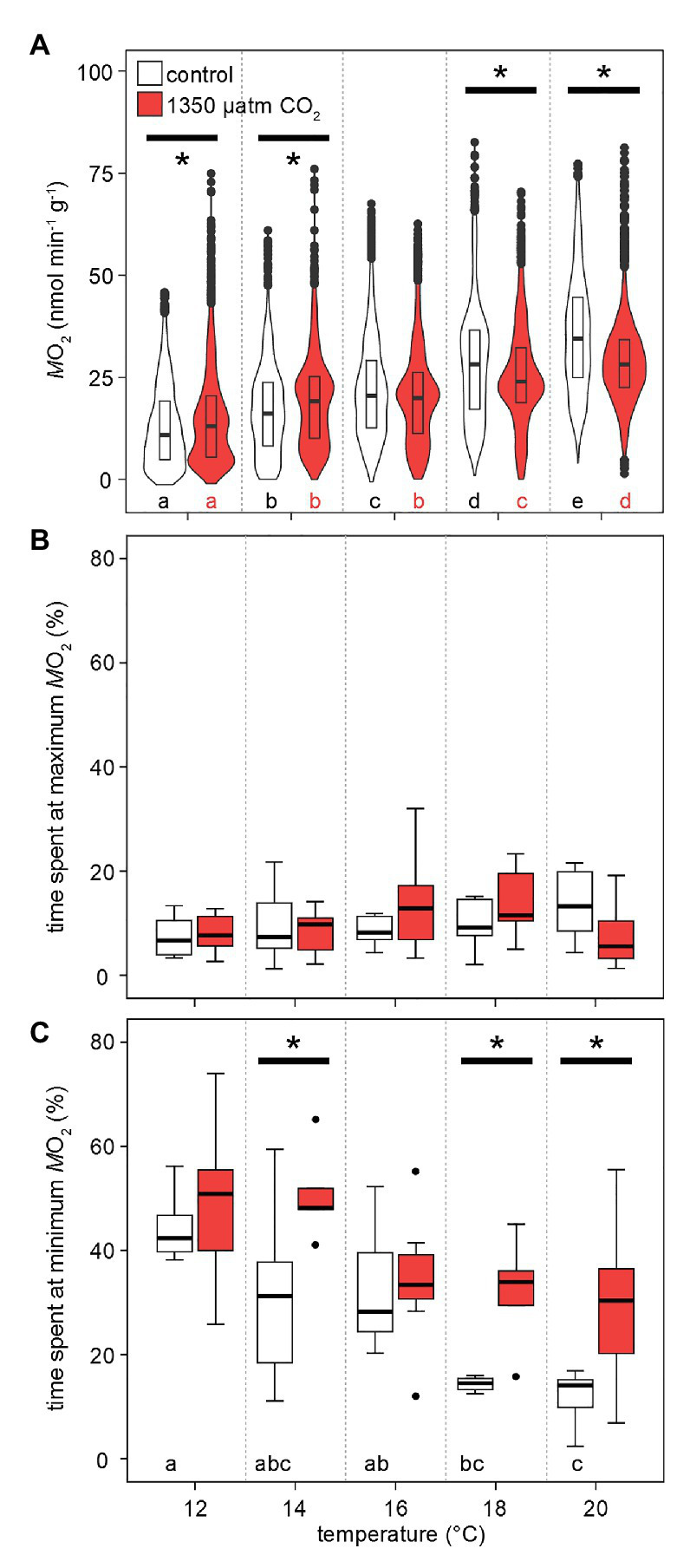
Effects of temperature and *P*_w_CO_2_ on metabolic rates. **(A)** Probability density distribution of metabolic rate (*n* = 4 from MRI experiments per *P*_w_CO_2_ + 3 from IR-PPG experiments). The maximum width of the violins is proportional to the overall sample size. For clarity, interquartile ranges (IQR, box) and medians (bold line) are added. Circles = data points larger than 1.5 × IQR. **(B)** Grouped time spent in the highest 25% and lowest 25% **(C)** of total $\dot{M}O_{2}$ amplitude during phases of fluctuating activity. These data are corrected for significant outliers (Iglewicz and Hoaglin’s robust test for multiple outliers, modified Z score ≥ 3.5). Whisker = 1.5 × interquartile range (IQR), box = IQR, bold line = median, circles = outlying data points. Asterisks denote significant differences between control and high CO_2_ conditions at the respective temperature **(A)**: Tukey’s HSD all-pairwise comparisons following a linear mixed model; **(B,C)**: Wilcoxon rank-sum test; *p* < 0.05. Letters show significant differences between each temperature step for a constant *P*_w_CO_2_ **(A)**: Tukey’s HSD all-pairwise comparisons following a linear mixed model; **(B,C)**: ANOVA on ranks, Tamhane T2; *p* < 0.05.

Figure 3 shows the distribution of values recorded for $\dot{M}O_{2}$ at specific temperatures and CO_2_ levels. Metabolic rates were significantly different between all temperatures and CO_2_ levels. At 12°C, crabs mostly displayed a low $\dot{M}O_{2}$ around 5–10 nmol min^−1^ g^−1^ (wider violin in this range of values), though, under high CO_2_ only, individual peak metabolic rates already reached 75 nmol min^−1^ g^−1^ (narrow violin in this range; Figure 4A). This maximum was not substantially surpassed but more frequently reached during subsequent exposures to higher temperatures (increased width at the violin’s top with rising temperatures). Phases of undetectable metabolic activity ($\dot{M}O_{2}$ = 0 nmol min^−1^ g^−1^) were recorded under normocapnia until 18°C. Under hypercapnic conditions, zero-levels in $\dot{M}O_{2}$ were even found at 20°C. Warming caused a rise in median $\dot{M}O_{2}$, but the median $\dot{M}O_{2}$ under hypercapnia at 18 and 20°C was lower than the normocapnic median. Furthermore, the most frequently measured $\dot{M}O_{2}$ (indicated by the widest part of the violins in Figure 4A) shifted to higher values, but similar to the medians, this shift was larger under normocapnic warming across the entire temperature range. Eventually, this resulted in a more even distribution of values to both sides of the median for 18 and 20°C. Significant distribution differences were found for nearly all combinations of temperature and *P*_w_CO_2_. Generally, $\dot{M}O_{2}$ appears to scale stronger with temperature under normocapnia than under hypercapnia.

The shift in the distribution of the most frequently measured metabolic rates becomes more obvious when looking at the share of $\dot{M}O_{2}$ values in the lowest and highest quarter of the entire range of values. This division translates into the time at the minimum and maximum 25% of values during the phases of fluctuating activity. Under both *P*_w_CO_2_ levels and across the entire temperature range, all animals spent ~15% of the measurement time in the top quarter of activity levels (Figure 4B). On the other hand, warming reduced the time spent at minimum activities from 40 (12°C) to 17% (20°C) under normocapnia. When exposed to high-CO_2_ warming, animals spent significantly more time (30–50%) at low activity levels. This means that at 18 and 20°C, the animals spent nearly twice as long in the low activity range under hypercapnia when compared to control CO_2_ levels (Figure 4C).

### Ventilatory Water Flow and Hemolymph Flow

**Figure 5:**
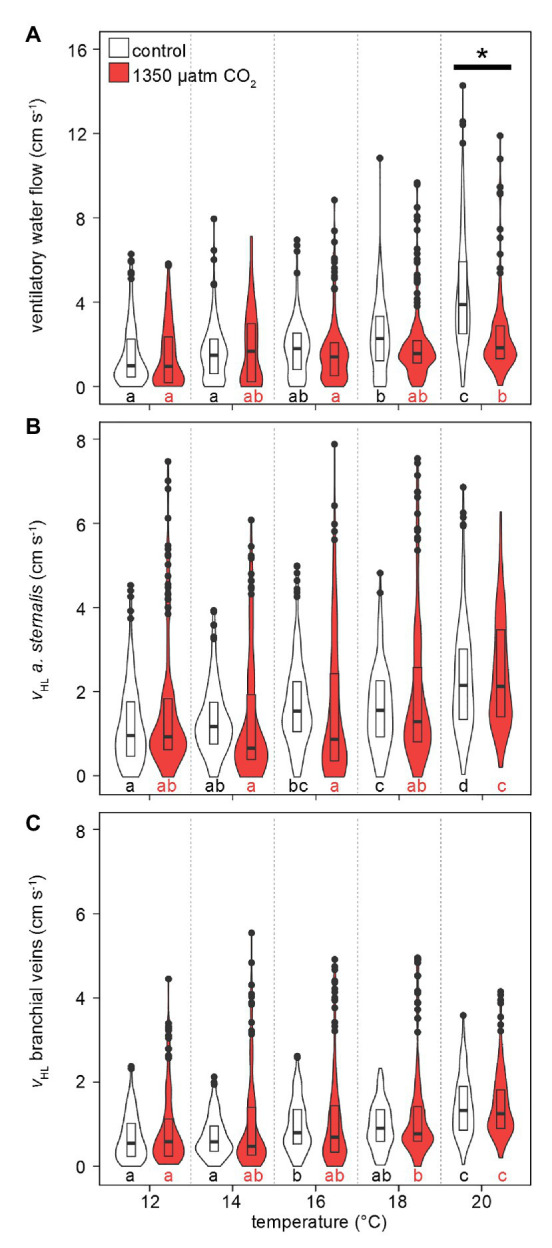
Effect of temperature and *P*_w_CO_2_ on ventilatory water flow and hemolymph flow velocity. **(A)** Ventilatory water flow through the branchial chamber. **(B)** Hemolymph flow velocity in the *arteria sternalis*. **(C)** Hemolymph flow velocity in efferent branchial veins, averaged for gills 6–8. Branchial water and hemolymph flow are presented here as sums for left and right gills, to account for shifts in unilateral gill utilization. Violins show probability density distributions of the respective parameters. The maximum width of the violins is proportional to the overall sample size (*n* = 4 animals per *P*_w_CO_2_). For clarity, interquartile ranges (IQR, box) and medians (bold line) are added. Circles = data points larger than 1.5 × IQR. Asterisks denote significant differences between control and high CO_2_ conditions at the respective temperature. Letters show significant differences between each temperature step for a constant *P*_w_CO_2_ (Tukey’s HSD all-pairwise comparisons following a linear mixed model; *p* < 0.05).

Like $\dot{M}O_{2}$, median and maximum ventilatory water flow and hemolymph flow increased with rising temperatures (Figure 5). Also, the maximum violin width shifted to higher values, indicating that higher ventilation and hemolymph flow velocities were measured more frequently with warming. Ventilatory water flow, recorded from velocity encoded phase-contrast MRI, revealed forward ventilation during virtually the entire set of measurements (negative values at the excurrent channel in Supplementary Figure S1). Toward the upper end of the temperature range, maximum ventilatory flow velocities were twice as high as at 12°C. Despite comparable peak velocities, hypercapnic conditions led to significantly lower ventilatory water flow at 20°C in comparison to normocapnic conditions (Figure 5A). A depressed ventilation under elevated CO_2_ is underlined by the nearly constant velocities recorded during hypercapnic warming, whereas velocities significantly increased during normocapnic warming between 16 and 20°C.

Warming caused an increase in hemolymph flow velocities in the *arteria sternalis* and the branchial veins (Figures 5B,C). Overall values were not significantly different between CO_2_ levels but maximum flow velocities were higher by ~2 cm s^−1^ under hypercapnia between 12 and 18°C. Despite the high maximum flow velocities under hypercapnia (up to 8 cm s^−1^ in the *arteria sternalis*), the most frequently measured flow velocities (widest part of the violin) between 12 and 16°C were found at relatively low values <2 cm s^−1^ or < 1 cm s^−1^ for the *arteria sternalis* or the branchial veins, respectively. At 18°C, the distribution of values becomes more similar for both CO_2_ levels in both vascular systems. At 20°C, the maximum flow velocities differed less between CO_2_ levels, signified by a decline in maximum flow velocities under high CO_2_. Consequently, both vascular systems showed nearly identical performance at 20°C, irrespective of *P*_w_CO_2_ (Figures 5B,C).

**Figure 6:**
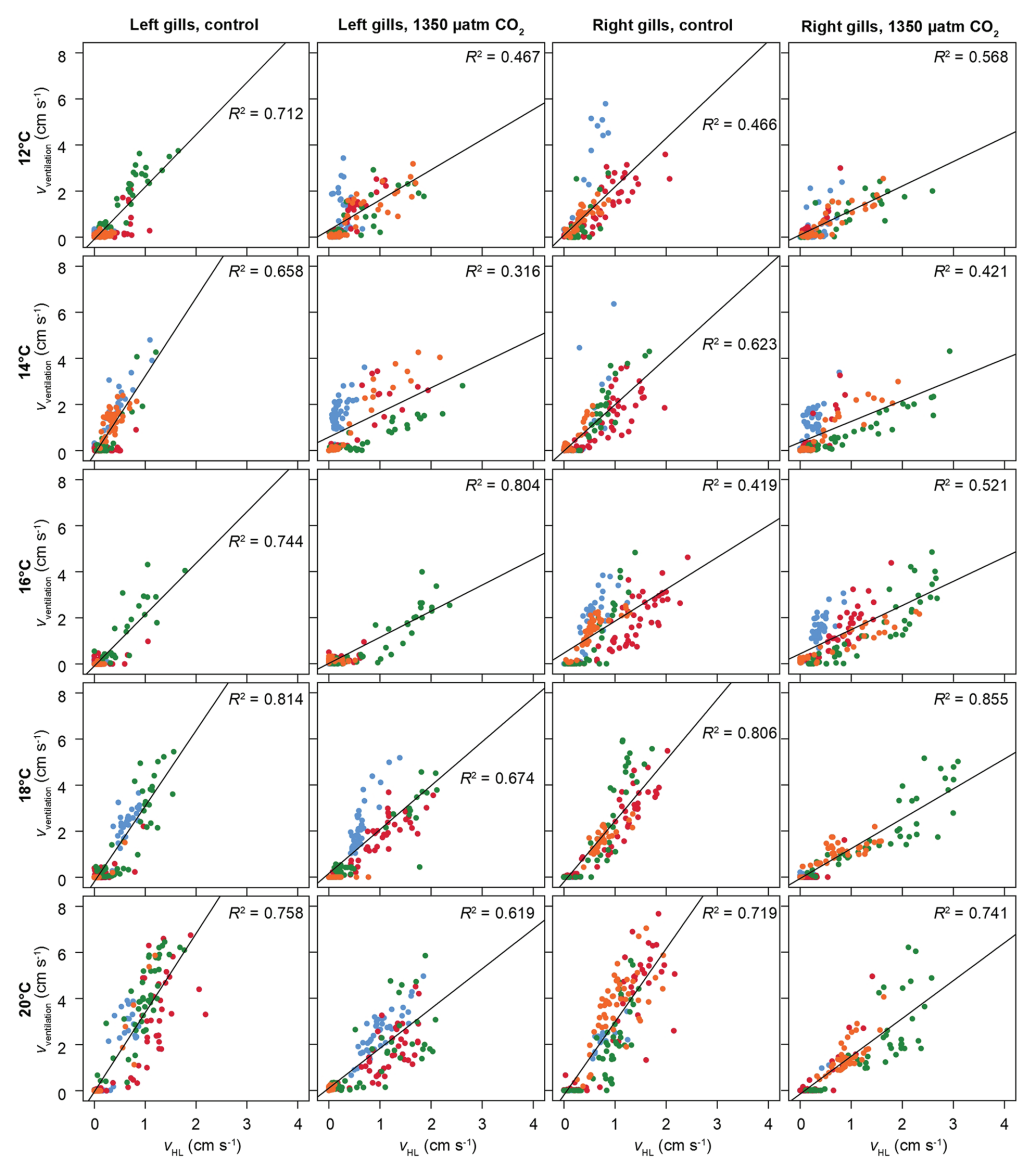
Correlation between hemolymph flow in branchial veins and ventilatory water flow. *v*_ventilation_ = velocity of ventilatory water flow. *v*_HL_ = velocity of hemolymph flow in branchial veins. Different colors show different animals. Flow velocities were recorded from coronal phase-contrast MRI (for scan parameters, see text). Correlations were separated for left and right gills. Squared linear correlations coefficients (Pearson) are given, together with a linear regression line for the entire set of animals per temperature and CO_2_ level. Significant positive correlations between hemolymph flow and water flow were found under all conditions (*p* < 0.05).

Figure 6 presents the correlation between ventilatory water flow and branchial hemolymph flow, separated for left and right gills, under all experimental conditions. There is a significant linear correlation between the two parameters. The lower ventilatory water flow under elevated CO_2_ in both gills (Figure 5A) is visible in a weaker correlation between ventilatory and hemolymph flow, resulting in a lower ventilation/perfusion ratio.

### Cardiac Performance

**Figure 7:**
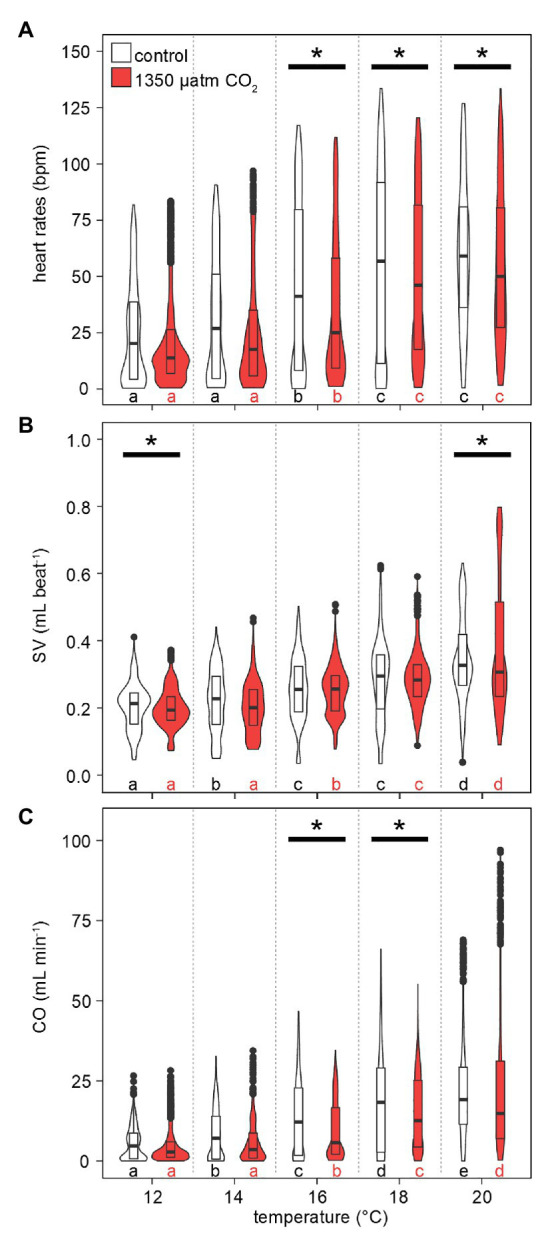
Effects of temperature and *P*_w_CO_2_ on cardiac performance. **(A)** Heart rates. **(B)** Stroke volume proxy, normalized to the 12°C mean of 0.2 ml under normocapnia and hypercapnia, respectively. **(C)** Cardiac output as a product of HR and SV. Violins show probability density distributions of the respective parameters. The maximum width of the violins is proportional to the overall sample size (*n* = 3 animals). For clarity, interquartile ranges (IQR, box) and medians (bold line) are added. Circles = data points larger than 1.5 × IQR. Asterisks denote significant differences between control and high CO_2_ conditions at the respective temperature. Letters show significant differences between each temperature step for a constant *P*_w_CO_2_ (Tukey’s HSD all-pairwise comparisons following a linear mixed model; *p* < 0.05).

The responses of heart rates to warming under both CO_2_ levels were similar to those observed for metabolic rates: lower HR were recorded more frequently at low-to-medium temperatures (maximum violin width between 0 and 25 bpm at 12–16°C, Figure 7A). This distribution gradually shifted to higher HR with rising temperatures, driven by rising maximum values and medians. Compared to $\dot{M}O_{2}$, the temperature-dependent increase in maximum HR values was delayed, as temperature-dependent differences were found in two steps: at 16°C and then at 18–20°C. At 12 and 14°C, maximum HRs were below 100 bpm. Highest HRs (~125 bpm) were found at 18°C and beyond. Hypercapnic HR medians were lower than normocapnic medians throughout, but this difference became significant only at *T*_w_ > 16°C, i.e., once the temperature effect on HR became significant. Conclusively, hypercapnia caused relatively depressed HR at the higher temperatures in this experiment.

**Figure 8:**
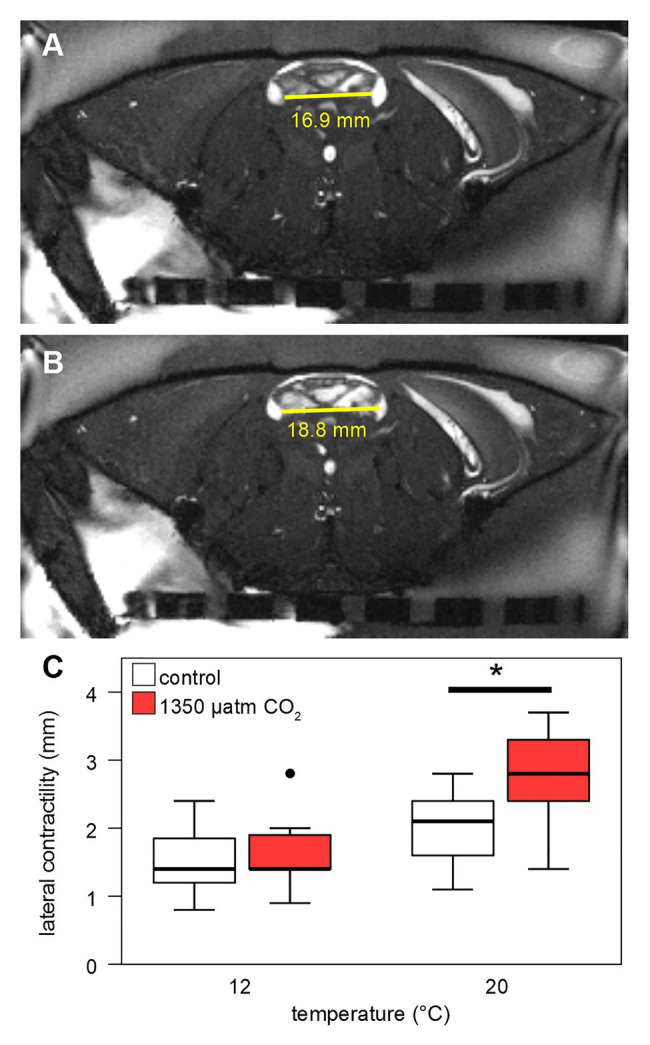
Contractility of the heart. Contractility was calculated from CINE MRI as difference between end-systolic **(A)** and end-diastolic **(B)** lateral diameter of the heart, in an axial slice including the two lateral ostia (endpoints of the horizontal yellow line). **(C)** Lateral contractility of the heart (in mm) is shown for 12 and 20°C control and high CO_2_-levels. Whisker = 1.5 × interquartile range (IQR), box = IQR, bold line = median, and circle = outlying data point. Hypercapnia caused a significant increase in contractility at 20°C (Student’s *t*-test, *p* < 0.05).

Stroke volumes (SV) under both CO_2_ levels were in the range of 0.05–0.4 ml at 12°C (Figure 7B). SVs differed significantly between normo‐ and hypercapnia at 12 and 20°C: Contrasting the slightly lower HR under elevated CO_2_ at 20°C, maximum hypercapnic SVs at 20°C were 0.2 ml higher, compared to maximum normocapnic SVs at this temperature. Furthermore, at 20°C the position of the third quartile was 0.1 ml higher under hypercapnia and while the minimum normocapnic SV remained below 0.1 ml during temperature increases, the minimum hypercapnic SV was higher throughout, surpassing 0.1 ml at 18°C. An increase in contractility (magnitude of heart contraction) under hypercapnic exposure at 20°C was confirmed by CINE MRI (Figure 8). Here, the lateral diameter of the heart changed by 2.8 ± 0.6 mm (13% of end-diastolic diameter) during contraction under hypercapnia and by 2.04 ± 0.55 mm (10% of end-diastolic diameter) under control CO_2_ levels (Figure 8). Both absolute and relative increases in contractility between low and high *P*_w_CO_2_ were significant (*t*-test; *p* < 0.05), supporting the idea of an increased SV at hypercapnic 20°C.

Maximum values for cardiac outputs (CO) increased continuously with rising temperatures for both normocapnic and hypercapnic conditions (Figure 7C). Again, a broad density distribution at low cardiac outputs between 12 and 16°C shifted to higher maximum and median values. Significant differences in CO under hypercapnia were found at 16 and 18°C. Following the trend in SV, hypercapnia delayed the temperature-dependent increase in CO, setting in only at 16°C and not already at 14°C (normocapnia). Still, the highest CO was recorded at hypercapnic 20°C, with values approaching 100 ml min^−1^, i.e., 25 ml min^−1^ higher than values at normocapnic 20°C.

## Discussion

This study investigated how elevated CO_2_ affects the temperature response of key components of respiratory and cardiac systems and how these parameters are differently affected by high ambient CO_2_ levels. A comparative, non-invasive multi-parameter approach was designed to better understand the mechanisms behind the previously reported enhanced thermal sensitivity under hypercapnia (Metzger et al., 2007; Walther et al., 2009).

Maximum habitat temperatures at the North Sea seafloor rarely exceed 20°C, but this temperature has been recorded during recent summers. Daily temperature fluctuations of 2–4°C were common in recent summers at 10 m water depth (Supplementary Figure S4). Given that the North Sea is projected to warm faster than 90% of the other oceans (Hobday and Pecl, 2014), maximum temperatures exceeding 20°C are likely to occur in the foreseeable future. Even under the temperature regime applied here, *C. pagurus* displayed limited ventilation and an altered mode of cardiac work if warming was combined with exposure to elevated *P*_w_CO_2_. Daily and especially seasonal fluctuations of *P*_w_CO_2_ are reported for the North Sea (Atamanchuk et al., 2014; Melzner et al., 2020). The present results highlight how a large decapod crustacean may respond to acute warming under different CO_2_ levels in its habitat.

### Methodology

The recorded values for *P*_w_CO_2_ and pH_w_ reflected the normocapnic and hypercapnic treatments, with consequences for physiological patterns. While significant differences in [HCO_3_^−^]_w_ were found in the IR-PPG experiments, they are an order of magnitude lower than what was reported to affect whole-animal activity (Maus et al., 2018). Overall, the variations in DIC and TA between treatments were similar to values reported in other studies (Lannig et al., 2010).

Due to the complexity of the measurements and limited animal availability at that time, the study is restricted to comparatively low animal numbers and an uneven inclusion of male and female crabs. The generality of the present results is thus limited, but a conclusive picture is still retrievable in the context of this work: animals showed similar behaviors during experiments in the MRI scanner and in the IR-PPG setup. Metabolic rates showed no differences at 12°C between repeated measurements on individuals or when comparing both setups. The continuous $\dot{M}O_{2}$ measurements revealed pausing behavior for about 80% of total experimental time across animals, indicating prevalent resting conditions in any setup. The results obtained by IR-PPG are supported well by separate MRI: CINE MRI determined a higher contractility under hypercapnic warming (corresponding to higher SV) and flow velocity measurements showed stable hemolymph flow patterns (corresponds to stable CO).

Simultaneous analyzes of multiple cardiovascular, ventilatory and metabolic parameters considerably limit the number of applicable non-invasive methods. For the present study, non-invasive measurements of restraint animals were selected because the confinement in a box with the limited ability to move its legs was considered as a minor disturbance for the animal compared to conventional invasive techniques. Effects of the fixation on the animals’ metabolic rate and cellular energy reserves have not been found in previous studies (Maus et al., 2018, 2019a,b); however, these studies did not investigate animals for more than 1–2 days. At 12°C, pausing behavior and unilateral ventilation persisted for 5 days (Supplementary Figure S5). While not obvious in these trials, prolonged starvation has been shown to lower metabolic rates (Ansell, 1973; Wallace, 1973). Considering the higher energy demand at higher temperatures and starvation theoretically decreasing the amplitude of $\dot{M}O_{2}$ cycles, the results at 20°C may be an underestimation depending on the degree of starvation. Separate experiments with long-term acclimated crabs at different temperatures and *P*_w_CO_2_ could help to identify the impact of starvation on the present data, also considering the negative impact of hypercapnia on feeding behavior and energy reserves of *C. pagurus* (Wang et al., 2018). Measurement times on acclimated crabs will also be shorter, reducing potential impacts of restraint on the cardiac responses (McGaw et al., 2018). Still, our results compare well to other reports on *in situ* HR (Ansell, 1973), including thermal responses of HR in large, unrestraint crustacea (Frederich and Pörtner, 2000).

Inconclusive results exist about the impact of sex on HR. While sex affected especially prolonged HR measurements, this impact was also shown to be minor compared to the effects of temperature, molting stage, and interindividual variability (Aagaard, 1996). This is in line with our results, where combined effects of temperature and CO_2_ have the largest impact on HR in our experiments (Supplementary Table S1). Follow-up experiments should nevertheless aim for a more balanced sex ratio.

To harmonize the variable routine activities of undisturbed crabs between setups and treatments, only phases of fluctuating activity were analyzed (pausing behavior; Ansell, 1973; McDonald et al., 1977,^1980^; Burnett and Bridges, 1981). The fluctuations in $\dot{M}O_{2}$ were comparable across individuals and setups under given conditions. Temporal changes in cardiovascular performance paralleled the patterns in $\dot{M}O_{2}$ and similar changes are also reported for hemolymph oxygen saturation (Burnett and Bridges, 1981; Frederich and Pörtner, 2000; Metzger et al., 2007). Three animals from the initial cohort showed non-fluctuating, stable performance levels, that were almost unaffected by temperature increases over an entire week of experiments. Such behavior was not related to feeding status, sex or size but displayed by animals with multiple missing appendages and regenerating limb buds. These animals were excluded from the present analysis, as regeneration of missing appendages affects metabolic rates (Skinner, 1962) and focusing on the pausing behavior was a key feature of this study.

Crabs modulate their ventilatory water current mainly through changes in scaphognathite stroke frequency, as the scaphognathite’s stroke volume is relatively constant for a given animal size (Cumberlidge and Uglow, 1977; Mercier and Wilkens, 1984). The detected changes in ventilatory water flow should, therefore, translate directly into changes in scaphognathite beat frequency and give an idea of oxygen supply capacities *via* ventilation (Batterton and Cameron, 1978; McDonald et al., 1980). Changes in the resistance of inhalant openings may also alter water flow through the gills, but should only shift the relationship between water flow and scaphognathite frequency and not its general interdependence.

The range of the cardiac stroke volume (SV) at 12°C control conditions was comparable to direct quantifications *via* MRI (Maus et al., 2019a), even after normalization to a mean SV of 0.2 ml. The maximum range of SV determined at 20°C was similar to values determined *via* the thermo-dilution technique and the Fick principle (Burnett et al., 1981; Bradford and Taylor, 1982). Values for weight-specific cardiac output (CO) lie in the range of those reported for *Maja squinado* and *Metacarcinus magister*, obtained with implanted Doppler flowmeters (De Wachter and McMahon, 1996; Frederich et al., 2000). The absolute accuracy of the CO depends on the accuracy of the stroke volume proxy (i.e., the plethysmograph signal), which in turn is affected by the position of the IR sensor on the carapace. Still, changes in SV and CO relative to a 12°C mean could be reliably derived for crabs exposed to either water CO_2_ level.

### Metabolic Activity

Whole-animal metabolic rates serve as a general integrator of animal activity. Pauses in oxygen consumption actually represent minimum systemic activity and the periodic peaks in $\dot{M}O_{2}$ were no experimental artifact elicited by the outwash of deoxygenated branchial water upon resumption of ventilation. This was previously confirmed in *C. pagurus* through direct measurements of the difference between post-branchial and pre-branchial *P*_e_O_2_. During ventilatory and metabolic pauses, this difference became zero and there were also no changes in the *P*O_2_ of branchial waters and hemolymph *P*CO_2_. Furthermore, no anaerobic metabolism was detected either during a pause (Burnett and Bridges, 1981). Shorter pauses as found under normocapnic warming might be the simple thermal response of a higher energy (and thus oxygen) demand under warming. Continuously long pauses, as under hypercapnic warming, would then result in a reduced capacity for aerobic performance over time. Since the spike in $\dot{M}O_{2}$ after a pause is no longer under hypercapnia, more net time is spent in a hypometabolic state with low hemolymph oxygen levels. While hemolymph *P*O_2_ was not measured here, a limited capacity for aerobic performance may indicate some degree of oxygen limitation when *C. pagurus* experience sub-critical warming under elevated CO_2_ levels. This, and the hypoventilation found under hypercapnia at 18–20°C, corroborate the results by Metzger et al. (2007), where severe hypercapnia led to reduced hemolymph oxygen levels at lower temperatures. The potentially narcotic effect of CO_2_ on whole-animal activity (prolonged phases of low $\dot{M}O_{2}$) is in line with the reduced foraging behavior displayed by *C. pagurus* after prolonged exposure to high CO_2_ levels (Wang et al., 2018). Phases of spontaneously elevated $\dot{M}O_{2}$ were not covered here but should also be considered in the future, since their scope is affected by seawater carbonate chemistry in *Carcinus maenas* even after prolonged exposure (Maus et al., 2018).

A functional correlation of $\dot{M}O_{2}$ with cardiovascular activities was already proposed by Ansell (1973). Periodic fluctuations in respiratory and cardiac activity at rest were later attributed to central nervous pacemakers regulating animal activity (Taylor, 1982). Combined with the results on metabolic rates, the present findings on cardiovascular and ventilatory performance contribute to a causal explanation of reduced hemolymph *P*O_2_ under hypercapnic warming.

### Ventilatory Performance and Cardiovascular Function

Exposure to elevated CO_2_ levels suppressed the warming-induced increase in ventilation, i.e., causing a relative hypoventilation compared to normocapnic warming at 18–20°C. In the branchial veins, which constitute the venous return in Decapoda, hemolymph flow velocities remained unaffected by hypercapnia and showed a thermal stimulation similar to normocapnia. A stable venous return is supported by a high cardiac output (CO). Impaired ventilation at stable cardiovascular performance (branchial perfusion and CO) was already reported for *Maja squinado*: There, it is indicative of normocapnic pejus temperatures, where temperature-dependent tissue oxygen demand cannot be met by the rate of ventilatory oxygen uptake (Frederich and Pörtner, 2000). A limited capacity for oxygen uptake due to a reduced ventilation/perfusion ratio may now explain the reduced *P*_e_O_2_ in the pericardial sinus of *C. pagurus* during rapid warming and severe hypercapnia (Metzger et al., 2007).

Since ventilation continued to increase with temperature under normocapnia, the normocapnic *T*_p_ (indicated by the onset of a temperature-insensitive ventilation), is most likely beyond the present-day maximum habitat temperatures of 20°C (Supplementary Figure S4) in the southern North Sea. A negative impact of hypercapnia on thermal tolerance is still implied here: while warming stimulated ventilation under normocapnia until 20°C, hypercapnia suppressed this stimulation, resulting in a significant hypoventilation at 20°C. Hypoventilation coincided with changes in cardiac work: slightly lower HRs under hypercapnia were offset by elevated SVs. Within the constraints placed by small numbers of experimental animals, *C. pagurus* appear to respond to hypercapnic warming with less-frequent but stronger heart beats, resulting in a net constant cardiac output. Based on relative hypoventilation, bradycardia, and the prolonged time spent in a low metabolic state (all compared to normocapnic values), we conclude that temperatures between 18 and 20°C may characterize the hypercapnic pejus temperatures for *C. pagurus* in the present experiments.

Pejus temperatures are the first indicators of thermal limitation and will be experienced by wild stocks more frequently than critical temperatures. Since limitations in aerobic performance beyond *T*_p_ translate into reduced growth and reduced scope for activity, sub-critical performance limitations may already prove detrimental for a population’s long-term abundance (Pörtner and Knust, 2007; Rutterford et al., 2015; Pörtner et al., 2017). Given that oceans take up excess CO_2_ from the atmosphere in an ongoing process called ocean acidification (Le Quéré et al., 2010), the present results underline the need for a more detailed understanding of the physiological implications of a high-CO_2_ world. This will be crucial for reasonable projections on ecosystem levels.

While the effect of CO_2_ on ventilation now contributes to explain reduced thermal tolerance under hypercapnia, the role of the cardiac muscle’s oxygen demand in shaping thermal sensitivity under hypercapnia (Walther et al., 2009) requires further investigation of the complex interplay of HR and SV in shaping CO (see section Introduction). Constant mean HR between 18 and 20°C under both hypercapnia or normocapnia could diminish the role for HR in defining CO between these temperatures. Compensating for (albeit slightly) reduced HR through elevated SV is a common response to hypoxia in Brachyura and the effect of hemolymph oxygen levels in setting cardiac work is well-described (Wilkens, 1993; Airriess and McMahon, 1994). While the cardiac responses to warming under hypercapnic conditions were not as severe as the responses to severe hypoxia, it appears that similar modifications in myocardial function are employed to maintain CO here. The limited oxygen uptake due to hypoventilation under hypercapnic warming causes hypoxemia, which in turn causes changes in cardiac work. Hypoxia-associated bradycardia is affected by reduced burst activity of the oxygen-sensitive cardiac ganglion (Wilkens, 1993). A concomitant increase in SV is less clearly explained but may be affected through combined neuronal and hormonal control (Wilkens and McMahon, 1992; McMahon, 2001). The effects may depend on the degree of hypoxemia, since the post-branchial *P*_e_O_2_ associated with bradycardia under severe hypoxia is reportedly lower than the *P*_e_O_2_ found at pejus temperatures in *C. pagurus* (Bradford and Taylor, 1982; Metzger et al., 2007). Even beyond direct effects of hypoxemia on the cardiac muscle, mitochondrial dysfunction can limit the capacity to increase HR in response to acute warming (Oellermann et al., 2020).

CO_2_ may yet have a superimposed effect on the heart of a decapod crustacean since hypercapnia caused tachycardia during acute warming (1°C h^−1^) in *Hyas araneus* (Walther et al., 2009). In any case, tachycardia or elevated SV should both maintain branchial perfusion as confirmed here, despite potential implications for the oxygen demand of the cardiac muscle. The different cardiac responses between 1°C h^−1^ warming in *H. araneus* (Walther et al., 2009) and 0.2°C h^−1^ warming in *C. pagurus* (present study) indicate complex relationships between CO_2_ exposure time, dose, and the rate of temperature change. Employing variable temperature and CO_2_ regimes is envisioned for future experiments to improve transferability to ecology and our general understanding of cardio-physiological plasticity in Decapoda.

Similar to the heart, the scaphognathites are sensitive to hemolymph oxygen levels but the critical *P*_e_O_2_ causing ventilation to fail is far below the *P*_e_O_2_ associated with *T*_p_ (Bradford and Taylor, 1982; Metzger et al., 2007). In the range of hypercapnic warming applied here, hypoxemia is an effect of low ventilation rather than its cause. It is worth noting that the post-branchial *P*_e_O_2_ associated with declining ventilation matches the *P*_e_O_2_ found at critical temperatures (*T*_c_) at ~2 kPa. At this *P*_e_O_2_, hemocyanin is no longer saturated, coinciding with breakpoints in $\dot{M}O_{2}$ and cardiac output and the onset of anaerobiosis (Bradford and Taylor, 1982; Metzger et al., 2007). Systemic oxygen limitation sets in at the *T*_c_ of *C. pagurus*, preceded by an already low hemolymph oxygen content following hypoventilation at *T*_p_.

Ventilation in water breathing crabs is mainly driven by the demand for oxygen. Metabolic CO_2_ is released to the water along with the diffusion gradient and due to its high solubility (Batterton and Cameron, 1978). High ventilation rates still improve CO_2_ diffusion to the water (Wheatly and Taylor, 1981), so hypoventilation in response to hypercapnia at elevated temperatures was an unexpected response. On the other hand, hypoventilation reduces the substantial costs for ventilation in a viscous medium (McDonald et al., 1980), allowing the animal to spend more time in a hypometabolic state (Figure 4C). Reducing aerobic costs for ventilation over time may be a transient response to relatively acute changes in water temperature and *P*CO_2_. The present results should be complemented by long-term studies to elucidate potential adaptation responses as the reduced oxygen uptake capacities may not be sustainable for extended periods of time.

It remains unclear if limited ventilation and the associated consequences of a reduced metabolic activity are a behavioral response to transient, unfavorable conditions, or a functional limitation under combined warming and hypercapnia. Functional limitations and shifts in ion exchange processes cause metabolic depression in invertebrates under adverse environmental conditions, including hypercapnia (Pörtner et al., 1998, 2000). Exposure to hypercapnic warming causes changes in extracellular ion concentration (Rastrick et al., 2014; Klymasz-Swartz et al., 2019). Considering the temperature-dependent permeability of ion channels (O’Leary and Marder, 2016), additional disruptions in ion and pH homeostasis following hypercapnic ion exchange potentially lead to a tipping point for neuronal function (Robertson and Money, 2012). Exposure to elevated CO_2_ levels and associated compensatory acid-base regulation may become intolerable at high temperatures. As a hypothesis, CO_2_-associated hypoventilation in the present study may be caused by neuronal malfunction due to shifting ion and pH gradients over neuron cell membranes. Osmoregulating crabs are able to withstand disturbances of ion and pH homeostasis during warming, so their systemic *T*_p_ is at higher temperatures compared to crabs with weak osmoregulation capacities, like *C. pagurus* (Jost et al., 2012). Further studies are required to characterize potential limitations of ion regulation mechanisms at *T*_p_.

The involvement of acid-base balance in setting thermal tolerance is supported by the fact that active acid-base regulation fails and temperature-dependent changes in pH_i_ and pH_e_ diverge to more alkaline values above *T*_p_ (Wood and Cameron, 1985; van Dijk et al., 1997; Mark et al., 2002). The additional exposure to hypercapnia induces non-linear deviations from the normocapnic temperature-dependent changes in body fluid pH *in vivo* (Zittier et al., 2013; Rastrick et al., 2014; Klymasz-Swartz et al., 2019).

Impaired neuronal functions may affect the ventilatory control more strongly than cardiac control because of the comparatively more complex structure of the network controlling ventilation (Taylor, 1982; Robertson and Money, 2012). Furthermore, the scaphognathite motion requires coordinated levator and depressor action to drive a ventilatory water current through the gills and this coordination could – in theory – be disturbed by ionic imbalances over the membranes of associated neurons. It is currently not known if these variable responses in acid-base regulation to warming and hypercapnia are species-dependent and thus functional or if they are defined by the experimental conditions.

### Conclusions and Perspectives

The present findings are the first to indicate a ventilatory response by an aquatic crustacean model to combined hypercapnia and warming. Due to its effect on oxygen uptake, limited ventilation may contribute to explain reduced thermal tolerance of a crustacean under the effects of CO_2_. Less frequent but stronger heart beats were confirmed by two non-invasive setups. They stabilize branchial perfusion but appear to be a response to reduced oxygen uptake that is initially caused by limited ventilation. To investigate the generality of the present results, a larger scale study is envisioned, where measurements also account for the animals’ characteristic pausing behavior. The observed responses to comparatively benign warming and hypercapnia are hidden within the changes of the animals’ routine activities. Fluctuating baseline activities with pronounced peaks and depressions cast doubt on the usefulness of mean values alone, as analyses of frequencies and patterns over time seem necessary. However, frequency changes are displayed to different degrees by different animals, complicating this approach. If hypoventilation in response to high-CO_2_ warming proves to be a general response among Brachyura, more detailed studies on the roles of ion and pH regulation in thermal tolerance are encouraged, too. The effects of CO_2_ on temperature-dependent acid-base regulation have thus far been tested only in a few studies and they may have an impact on neuronal functions setting systemic responses.

## Data Availability Statement

The datasets generated for this study can be found in the public repository PANGAEA: doi.pangaea.de/10.1594/PANGAEA.924119.

## Ethics Statement

Ethical review and approval was not required for the animal study because experiments were performed on crustaceans.

## Author Contributions

BM, CB, and HOP conceived the study design and experimental setup. BM and SG performed the experiments and analyzed the data. All authors interpreted the data, wrote the manuscript, and approved of its final version.

### Conflict of Interest

The authors declare that the research was conducted in the absence of any commercial or financial relationships that could be construed as a potential conflict of interest.

## Abbreviations

CINE: Cinematography
CO: Cardiac output
DIC: Dissolved inorganic carbon
FR: Flow rate
HR: Heart rate
IR-PPG: Infrared photoplethysmography
$\dot{M}O_{2}$: Whole-animal metabolic rate
MRI: Magnetic resonance imaging
*P*_e_O_2_: Extracellular/hemolymph partial pressure of oxygen
*P*_w_CO_2_: Partial pressure of CO_2_ in water
SV: Stroke volume
SVP: Stroke volume proxy
TA: Total seawater alkalinity
*T*_c_: Critical temperature
*T*_p_: Pejus temperature
*T*_w_: Water temperature
*w*_f_: Fresh weight
