# Supplementary material for: Non-invasive MRI Studies of Ventilatory and Cardiovascular Performance in Edible Crabs Cancer pagurus During Warming Under Elevated CO2 Levels
Source: Front Physiol. 2021 Jan 11;11:596529. doi: 10.3389/fphys.2020.596529 (PMC7831881; doi:10.3389/fphys.2020.596529)
Supplement: Supplementary file 1 [file Table_1.docx]

# Supplementary Material

Non-invasive MRI Studies of Ventilatory and Cardiovascular Performance in Edible Crabs *Cancer pagurus* During Warming Under Elevated CO_2_ Levels

**Figures**


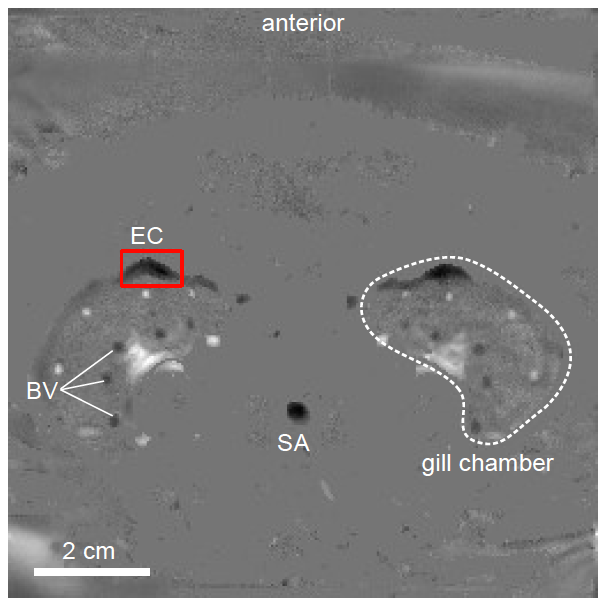


**Fig. S1 Flow-encoded phase-contrast MR image of *C. pagurus*.** Dorsal view, coronal orientation. The image gives quantitative information of flow velocities in each pixel (black = negative value, flow towards ventral; white = positive value, flow towards dorsal). The gills and gill chambers are visible laterally. Branchial veins (BV) are divided into an ascending outer part (white) and descending inner part (black). Mean branchial haemolymph flow velocities were measured in the three posterior inner BV for both left and right gills and in the sternal artery (SA). The excurrent channel (EC) is highlighted for the left gill chamber.


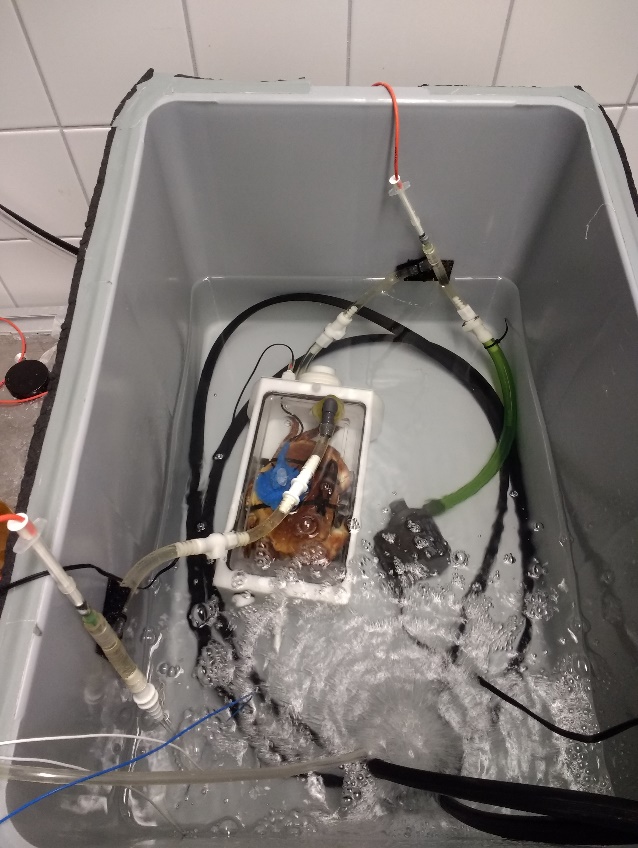


**3**

**6**

**7**

**5**

**4**

**2**

**1**

**Fig. S2 Experimental setup of IR-PPG experiments.** Order of numbers 1-4 represent the direction of water flow through the setup. The chamber was submerged in seawater. At the pump (maximum performance of 490 L/h; Eheim GmbH & Co. KG, Deizisau, Germany) (1), the water entered the circulation and passed the inlet-optode (2). From there, it entered the chamber containing the crab (3) with the attached photo-plethysmograph fixed dorsally with dental wax (6, blue). From there, the water passed the outlet-optode (4) and exited the hose system (all hoses: inner diameter of 0.6 cm; Tygon S3 E3603 meets NSF-51 STD; Saint-Gobain, France). Set levels of *P*_w_O_2_ and *P*_w_CO_2_ were maintained using an aeration stone (5). Water temperature was controlled by using thermic hoses connected to a thermostat (Lauda Eco RE 630; LAUDA DR. R. WOBSER GmbH & Co. KG, Germany) (7). During the experiments, the setup was covered by a lid to prevent visual disturbance of the animal and to minimize gas exchange.

**
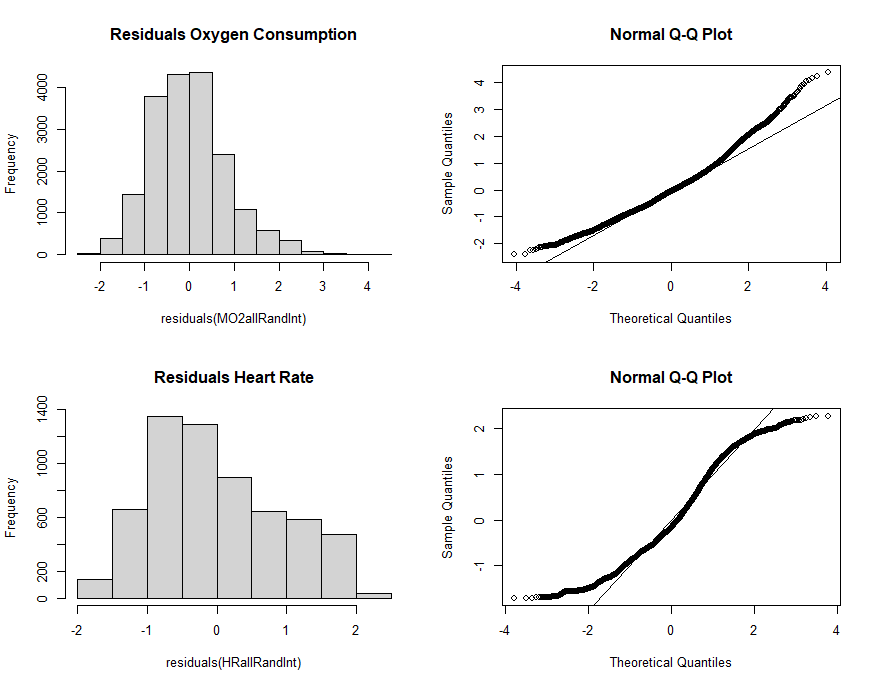

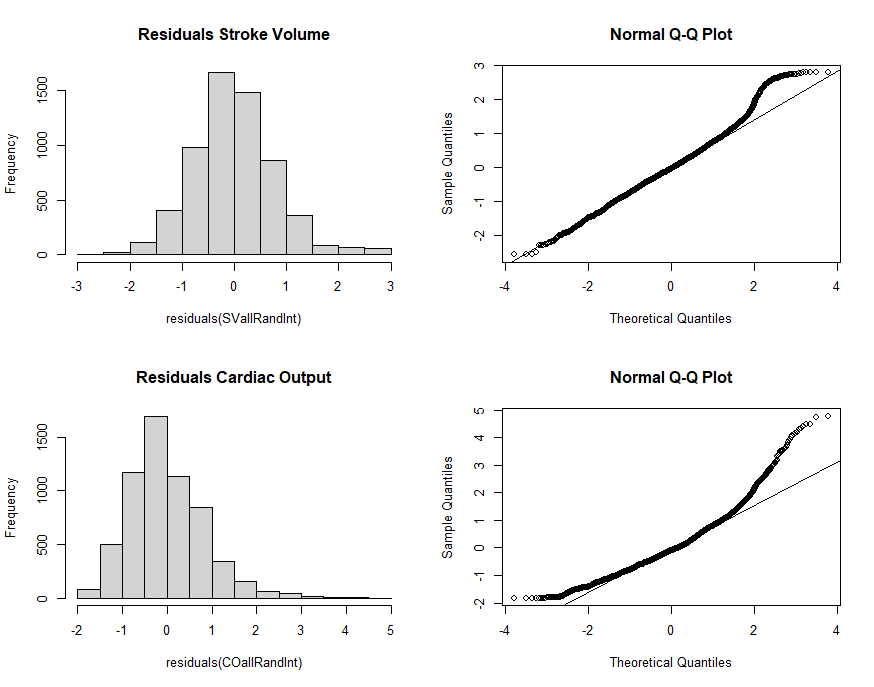
Fig. S3. Histograms (left) and corresponding Normal Q-Q plots (right) of the investigated parameters.** Given the large amount of observations, the overall robustness of the model was considered sufficient despite partial deviations from homoscedasticity.

**
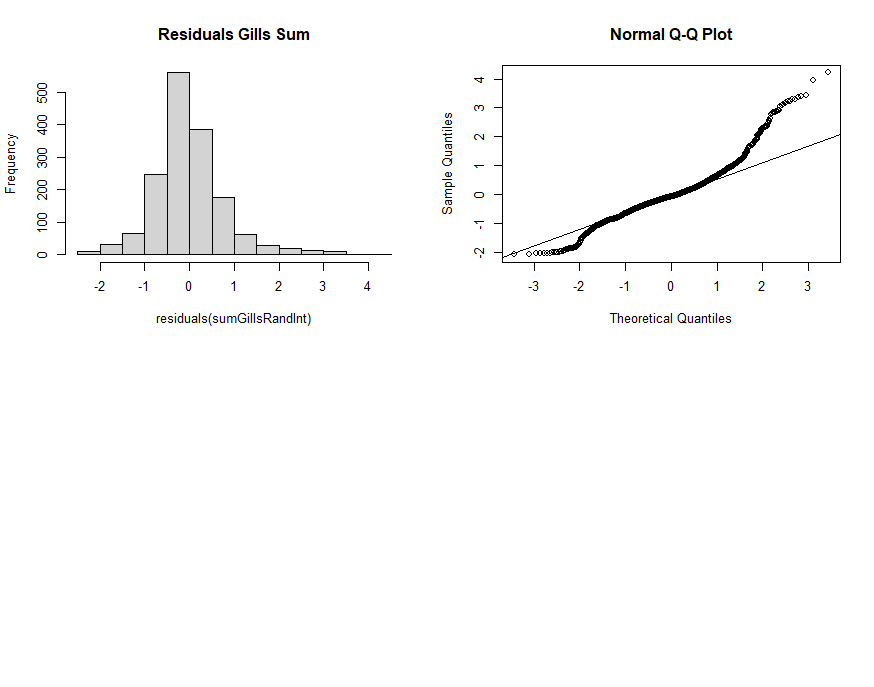

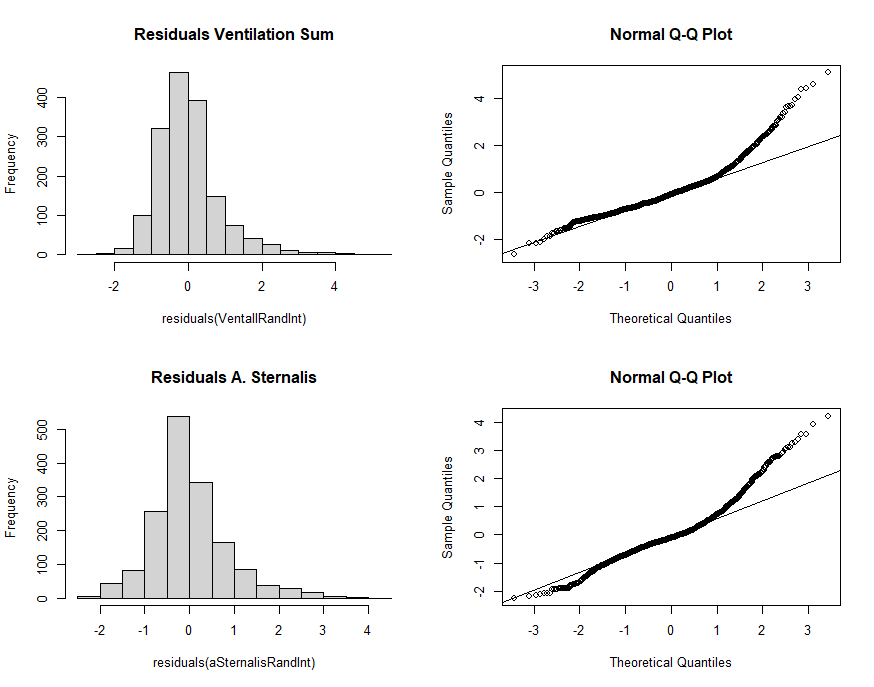

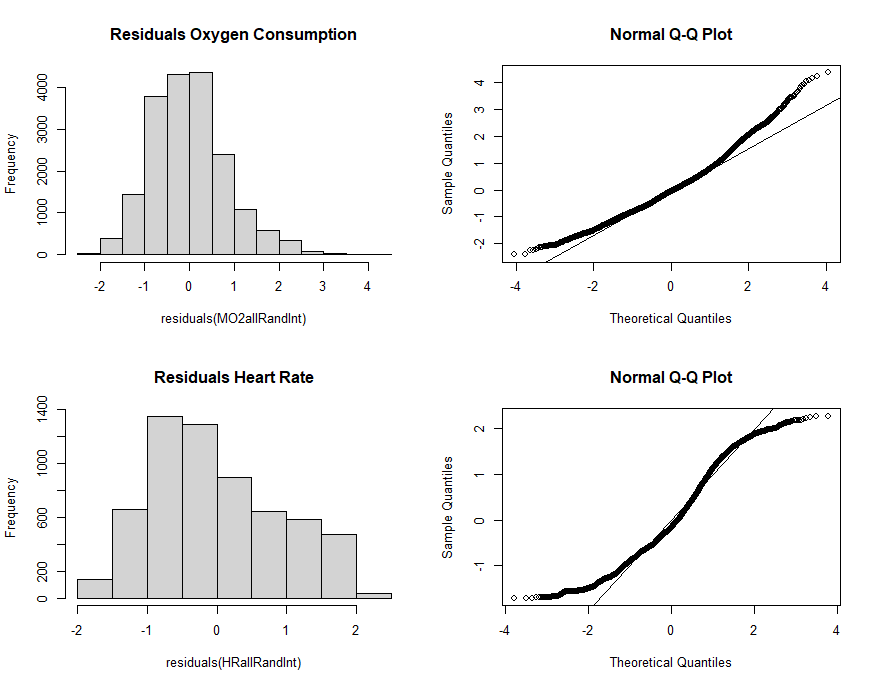
Fig S3 Continued.**


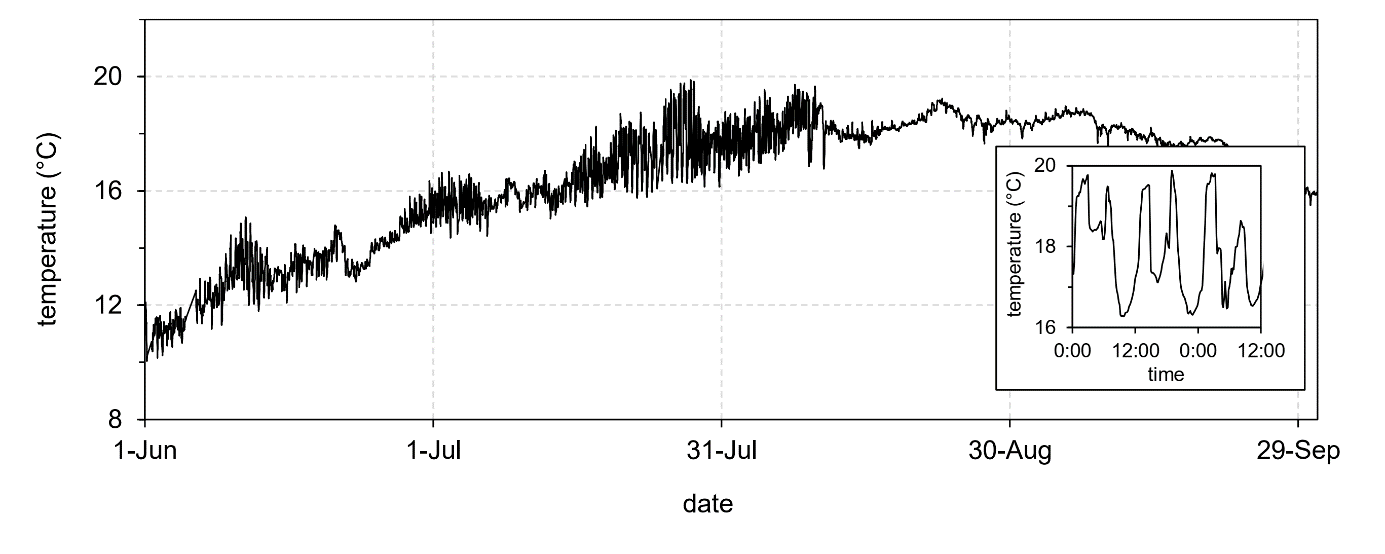
**Fig. S4 *In situ* water temperature record from the North Sea seafloor.** Data were recorded from June 1^st^ – September 30^th^ 2018 in 10 m depth at the Underwaternode Helgoland, provided by the AWI and HZG (<http://codm.hzg.de/codm/>). Location of the node is: 54.193°N; 7.876°E. The insert highlights the daily changes in temperature between the 27.7.2018 and the 28.7.2018.

**Fig. S5 Example of a time course of changes in metabolic rate at constant temperatures.** A 280 g female crab was placed in the setup for MRI experiments inside the magnet. Metabolic rate was continuously recorded in a flow-through setup at a rate of 1 min^-1^. Temperature was kept constant at 12°C and normocapnic conditions were set.

**Tables**

**Tab. S1 Results of likelihood-ratio-tests of the full model against reduced models.** The full model included Temperature, Condition and the interaction of Temperature and Condition as fixed effects. The reduced models contained either solely Temperature, solely Condition or both without interaction. Random effect structures with random intercepts per animal remained the same for all models. Results show *p*-values (significance level *p* < 0.05) for tests of the corresponding reduced model versus the full model. *p* <0.05 indicates significant improvement of the full model compared to the reduced model. **p*<0.05; ***p*<0.01; ****p*<0.001.

|  | Temperature | Condition | Temperature + Condition |
| --- | --- | --- | --- |
| Heart rate | 6.821179e-11*** | 3.785804e-198*** | 0.09723757 |
| Stroke Volume | 7.628267e-18*** | 0*** | 2.244931e-16*** |
| Cardiac Output | 9.93357e-17*** | 0*** | 1.439807e-12*** |
| Metabolic Rate | 6.62877e-76*** | 0*** | 3.807661e-76*** |
| Sum Ventilation | 2.07972e-22*** | 1.608372e-66*** | 3.963903e-19*** |
| A. sternalis | 0.01238567* | 9.68261e-30*** | 0.005824098 |
| Sum Gills | 0.08056244 | 4.764604e-30*** | 0.05068333 |

**Tab. S2 Results of the selected linear mixed effects model with random intercepts for each animal.** Values for the fixed effects at the corresponding dependent variable describe the slope compared to control conditions (“Constant”). Values in brackets describe the standard errors. Asterisks indicate difference of coefficients from zero. ‘:’ indicates investigation of the interaction between the fixed effects. Data were standardized prior to model testing.

*Dependent variable:*

Heart Rate Stroke Volume Cardiac Output Metabolic Rate Sum Ventilation A. sternalis Sum Gills

|  | (1) | (2) | (3) | (4) | (5) | (6) | (7) |
| --- | --- | --- | --- | --- | --- | --- | --- |
| high CO_2_ (1350 µatm) | -0.073 | 0.255^***^ | 0.106^*^ | 0.258^***^ | 0.019 | 0.201 | 0.101 |
|  | (0.052) | (0.045) | (0.049) | (0.029) | (0.102) | (0.102) | (0.097) |
| 14°C | 0.185^**^ | 0.315^***^ | 0.297^***^ | 0.328^***^ | 0.066 | 0.117 | 0.049 |
|  | (0.067) | (0.058) | (0.063) | (0.031) | (0.096) | (0.096) | (0.091) |
| 16°C | 0.608^***^ | 0.736^***^ | 0.729^***^ | 0.713^***^ | 0.192 | 0.411^***^ | 0.345^***^ |
|  | (0.055) | (0.048) | (0.052) | (0.030) | (0.099) | (0.099) | (0.093) |
| 18°C | 0.892^***^ | 0.862^***^ | 1.021^***^ | 1.113^***^ | 0.373^***^ | 0.278^**^ | 0.285^**^ |
|  | (0.054) | (0.047) | (0.051) | (0.030) | (0.099) | (0.099) | (0.093) |
| 20°C | 1.025^***^ | 1.343^***^ | 1.273^***^ | 1.632^***^ | 1.475^***^ | 0.772^***^ | 0.849^***^ |
|  | (0.054) | (0.047) | (0.051) | (0.030) | (0.096) | (0.096) | (0.090) |
| 14°C : high CO_2_ | -0.028 | -0.418^***^ | -0.265^**^ | -0.014 | 0.151 | -0.304^*^ | 0.064 |
|  | (0.090) | (0.078) | (0.084) | (0.042) | (0.141) | (0.141) | (0.133) |
| 16°C : high CO_2_ | -0.201^**^ | -0.391^***^ | -0.424^***^ | -0.322^***^ | -0.133 | -0.446^**^ | -0.173 |
|  | (0.073) | (0.064) | (0.069) | (0.040) | (0.140) | (0.140) | (0.132) |
| 18°C : high CO_2_ | -0.101 | -0.300^***^ | -0.387^***^ | -0.362^***^ | -0.131 | -0.038 | 0.019 |
|  | (0.076) | (0.066) | (0.071) | (0.040) | (0.138) | (0.138) | (0.131) |
| 20°C : high CO_2_ | -0.097 | 0.005 | -0.064 | -0.635^***^ | -1.028^***^ | -0.157 | -0.260^*^ |
|  | (0.070) | (0.061) | (0.066) | (0.039) | (0.137) | (0.137) | (0.130) |
| Constant | -0.507^***^ | -0.797^**^ | -0.697^***^ | -0.731^***^ | -0.253 | -0.220 | -0.193 |
|  | (0.046) | (0.278) | (0.164) | (0.099) | (0.143) | (0.237) | (0.291) |
| Observations | 6087 | 6087 | 6087 | 18854 | 1618 | 1618 | 1618 |
| Log Likelihood | -8139.989 | -7270.715 | -7746.455 | -23813.710 | -2090.908 | -2091.333 -2002.955 | |
| Akaike Inf. Crit. | 16303.980 | 14565.430 | 15516.910 | 47651.420 | 4205.816 | 4206.666 4029.911 | |
| Bayesian Inf. Crit. | 16384.550 | 14646.000 | 15597.480 | 47745.550 | 4270.483 | 4271.334 4094.578 | |

*Note:* ^*^p<0.05; ^**^p<0.01; ^***^p<0.001

**Tab S3. Results of post-hoc pairwise comparisons of treatments following linear mixed models for each parameter.** ‘Estimate’ describes the estimated marginal means; ‘SE’ the standard errors, ‘df’ the degrees of freedom, calculated via Kenward-Roger; *p*-values are obtained and adjusted for multiple comparisons via Tukey HSD, significance level *p*<0.05.

**Heart Rate**

**Comparisons Estimate SE df t-ratio *p*-value**

control 12 - OA 12 0.0734 0.0527 4448 1.394 0.9294

control 12 - control 14 -0.1846 0.0673 5759 -2.742 0.1573

control 12 - OA 14 -0.0833 0.0655 5954 -1.271 0.9601

control 12 - control 16 -0.6083 0.0558 3464 -10.897 <.0001

control 12 - OA 16 -0.3339 0.0559 4907 -5.968 <.0001

control 12 - control 18 -0.8919 0.0546 5067 -16.323 <.0001

control 12 - OA 18 -0.7171 0.0592 5980 -12.111 <.0001

control 12 - control 20 -1.0247 0.0545 3904 -18.800 <.0001

control 12 - OA 20 -0.8538 0.0530 5002 -16.096 <.0001

OA 12 - control 14 -0.2580 0.0622 4629 -4.147 0.0014

OA 12 - OA 14 -0.1567 0.0604 4828 -2.595 0.2202

OA 12 - control 16 -0.6817 0.0470 6064 -14.519 <.0001

OA 12 - OA 16 -0.4073 0.0480 6066 -8.488 <.0001

OA 12 - control 18 -0.9653 0.0465 6076 -20.767 <.0001

OA 12 - OA 18 -0.7905 0.0526 6006 -15.025 <.0001

OA 12 - control 20 -1.0982 0.0458 6038 -23.952 <.0001

OA 12 - OA 20 -0.9273 0.0446 6077 -20.783 <.0001

control 14 - OA 14 0.1013 0.0728 6077 1.391 0.9303

control 14 - control 16 -0.4237 0.0650 3650 -6.514 <.0001

control 14 - OA 16 -0.1493 0.0655 4227 -2.279 0.4028

control 14 - control 18 -0.7073 0.0640 4954 -11.056 <.0001

control 14 - OA 18 -0.5325 0.0680 5734 -7.834 <.0001

control 14 - control 20 -0.8402 0.0643 3460 -13.063 <.0001

control 14 - OA 20 -0.6693 0.0625 5082 -10.709 <.0001

OA 14 - control 16 -0.5250 0.0633 3818 -8.297 <.0001

OA 14 - OA 16 -0.2506 0.0637 4551 -3.936 0.0034

OA 14 - control 18 -0.8086 0.0622 5177 -13.003 <.0001

OA 14 - OA 18 -0.6338 0.0663 5890 -9.562 <.0001

OA 14 - control 20 -0.9414 0.0624 3735 -15.078 <.0001

OA 14 - OA 20 -0.7705 0.0607 5277 -12.696 <.0001

control 16 - OA 16 0.2744 0.0508 6053 5.405 <.0001

control 16 - control 18 -0.2836 0.0495 6039 -5.735 <.0001

control 16 - OA 18 -0.1088 0.0555 5649 -1.962 0.6259

control 16 - control 20 -0.4165 0.0486 6073 -8.566 <.0001

control 16 - OA 20 -0.2456 0.0477 6004 -5.143 <.0001

OA 16 - control 18 -0.5580 0.0503 6073 -11.084 <.0001

OA 16 - OA 18 -0.3832 0.0560 6006 -6.841 <.0001

OA 16 - control 20 -0.6909 0.0496 6077 -13.938 <.0001

OA 16 - OA 20 -0.5200 0.0487 6057 -10.681 <.0001

control 18 - OA 18 0.1748 0.0547 6060 3.195 0.0457

control 18 - control 20 -0.1328 0.0484 6037 -2.747 0.1553

control 18 - OA 20 0.0381 0.0472 6076 0.807 0.9985

OA 18 - control 20 -0.3076 0.0544 5763 -5.657 <.0001

OA 18 - OA 20 -0.1367 0.0531 6061 -2.574 0.2301

control 20 - OA 20 0.1709 0.0466 5973 3.664 0.0094

**Stroke Volume**

**Comparisons Estimate SE df t-ratio *p*-value**

control 12 - OA 12 -0.2550 0.0454 6076 -5.617 <.0001

control 12 - control 14 -0.3154 0.0581 6075 -5.425 <.0001

control 12 - OA 14 -0.1519 0.0567 6075 -2.682 0.1813

control 12 - control 16 -0.7360 0.0480 6076 -15.333 <.0001

control 12 - OA 16 -0.5996 0.0483 6076 -12.426 <.0001

control 12 - control 18 -0.8615 0.0471 6075 -18.277 <.0001

control 12 - OA 18 -0.8166 0.0512 6075 -15.945 <.0001

control 12 - control 20 -1.3432 0.0469 6076 -28.624 <.0001

control 12 - OA 20 -1.6033 0.0458 6076 -35.043 <.0001

OA 12 - control 14 -0.0605 0.0536 6076 -1.127 0.9820

OA 12 - OA 14 0.1030 0.0521 6076 1.978 0.6146

OA 12 - control 16 -0.4810 0.0406 6075 -11.837 <.0001

OA 12 - OA 16 -0.3446 0.0415 6075 -8.298 <.0001

OA 12 - control 18 -0.6066 0.0402 6075 -15.071 <.0001

OA 12 - OA 18 -0.5616 0.0455 6075 -12.339 <.0001

OA 12 - control 20 -1.0882 0.0397 6075 -27.434 <.0001

OA 12 - OA 20 -1.3483 0.0386 6075 -34.902 <.0001

control 14 - OA 14 0.1635 0.0631 6075 2.593 0.2209

control 14 - control 16 -0.4206 0.0560 6076 -7.515 <.0001

control 14 - OA 16 -0.2842 0.0564 6076 -5.037 <.0001

control 14 - control 18 -0.5461 0.0552 6076 -9.897 <.0001

control 14 - OA 18 -0.5012 0.0587 6075 -8.533 <.0001

control 14 - control 20 -1.0278 0.0553 6076 -18.585 <.0001

control 14 - OA 20 -1.2879 0.0539 6075 -23.886 <.0001

OA 14 - control 16 -0.5841 0.0545 6076 -10.725 <.0001

OA 14 - OA 16 -0.4477 0.0549 6076 -8.159 <.0001

OA 14 - control 18 -0.7096 0.0537 6075 -13.223 <.0001

OA 14 - OA 18 -0.6647 0.0573 6075 -11.598 <.0001

OA 14 - control 20 -1.1913 0.0537 6076 -22.174 <.0001

OA 14 - OA 20 -1.4514 0.0524 6075 -27.704 <.0001

control 16 - OA 16 0.1364 0.0439 6075 3.105 0.0597

control 16 - control 18 -0.1256 0.0428 6075 -2.934 0.0966

control 16 - OA 18 -0.0806 0.0479 6075 -1.682 0.8060

control 16 - control 20 -0.6072 0.0421 6075 -14.428 <.0001

control 16 - OA 20 -0.8673 0.0413 6075 -20.999 <.0001

OA 16 - control 18 -0.2620 0.0436 6075 -6.011 <.0001

OA 16 - OA 18 -0.2170 0.0485 6075 -4.478 0.0003

OA 16 - control 20 -0.7436 0.0429 6075 -17.328 <.0001

OA 16 - OA 20 -1.0037 0.0421 6075 -23.827 <.0001

control 18 - OA 18 0.0449 0.0473 6075 0.949 0.9948

control 18 - control 20 -0.4816 0.0418 6075 -11.512 <.0001

control 18 - OA 20 -0.7418 0.0408 6075 -18.163 <.0001

OA 18 - control 20 -0.5266 0.0470 6075 -11.204 <.0001

OA 18 - OA 20 -0.7867 0.0460 6075 -17.113 <.0001

control 20 - OA 20 -0.2601 0.0403 6075 -6.449 <.0001

**Cardiac Output**

**Comparisons Estimate SE df t-ratio *p*-value**

control 12 - OA 12 -0.1063 0.0491 6077 -2.164 0.4818

control 12 - control 14 -0.2972 0.0629 6076 -4.725 0.0001

control 12 - OA 14 -0.1389 0.0613 6076 -2.267 0.4109

control 12 - control 16 -0.7293 0.0519 6077 -14.045 <.0001

control 12 - OA 16 -0.4114 0.0522 6077 -7.883 <.0001

control 12 - control 18 -1.0211 0.0510 6076 -20.024 <.0001

control 12 - OA 18 -0.7400 0.0554 6076 -13.358 <.0001

control 12 - control 20 -1.2727 0.0508 6077 -25.071 <.0001

control 12 - OA 20 -1.3153 0.0495 6076 -26.575 <.0001

OA 12 - control 14 -0.1909 0.0580 6077 -3.291 0.0339

OA 12 - OA 14 -0.0327 0.0563 6077 -0.580 0.9999

OA 12 - control 16 -0.6230 0.0440 6075 -14.173 <.0001

OA 12 - OA 16 -0.3052 0.0449 6075 -6.794 <.0001

OA 12 - control 18 -0.9148 0.0435 6075 -21.013 <.0001

OA 12 - OA 18 -0.6337 0.0492 6075 -12.872 <.0001

OA 12 - control 20 -1.1664 0.0429 6075 -27.184 <.0001

OA 12 - OA 20 -1.2090 0.0418 6075 -28.932 <.0001

control 14 - OA 14 0.1583 0.0682 6075 2.320 0.3757

control 14 - control 16 -0.4321 0.0605 6077 -7.138 <.0001

control 14 - OA 16 -0.1143 0.0610 6077 -1.872 0.6880

control 14 - control 18 -0.7239 0.0597 6076 -12.128 <.0001

control 14 - OA 18 -0.4428 0.0635 6076 -6.970 <.0001

control 14 - control 20 -0.9755 0.0598 6077 -16.307 <.0001

control 14 - OA 20 -1.0181 0.0583 6076 -17.456 <.0001

OA 14 - control 16 -0.5903 0.0589 6077 -10.021 <.0001

OA 14 - OA 16 -0.2725 0.0594 6077 -4.591 0.0002

OA 14 - control 18 -0.8821 0.0581 6076 -15.196 <.0001

OA 14 - OA 18 -0.6010 0.0620 6076 -9.696 <.0001

OA 14 - control 20 -1.1337 0.0581 6077 -19.508 <.0001

OA 14 - OA 20 -1.1763 0.0567 6076 -20.758 <.0001

control 16 - OA 16 0.3178 0.0475 6075 6.689 <.0001

control 16 - control 18 -0.2918 0.0463 6075 -6.303 <.0001

control 16 - OA 18 -0.0107 0.0518 6076 -0.206 1.0000

control 16 - control 20 -0.5434 0.0455 6075 -11.937 <.0001

control 16 - OA 20 -0.5860 0.0447 6075 -13.116 <.0001

OA 16 - control 18 -0.6096 0.0471 6075 -12.933 <.0001

OA 16 - OA 18 -0.3285 0.0524 6075 -6.268 <.0001

OA 16 - control 20 -0.8612 0.0464 6075 -18.554 <.0001

OA 16 - OA 20 -0.9038 0.0456 6075 -19.835 <.0001

control 18 - OA 18 0.2811 0.0512 6075 5.488 <.0001

control 18 - control 20 -0.2516 0.0453 6075 -5.560 <.0001

control 18 - OA 20 -0.2942 0.0442 6075 -6.660 <.0001

OA 18 - control 20 -0.5327 0.0508 6076 -10.478 <.0001

OA 18 - OA 20 -0.5753 0.0497 6075 -11.569 <.0001

control 20 - OA 20 -0.0426 0.0436 6076 -0.976 0.9936

**Metabolic Rate**

**Comparisons Estimate SE df t-ratio *p*-value**

control 12 - OA 12 -0.2580 0.0293 18844 -8.815 <.0001

control 12 - control 14 -0.3282 0.0314 18838 -10.464 <.0001

control 12 - OA 14 -0.5722 0.0305 18844 -18.782 <.0001

control 12 - control 16 -0.7132 0.0298 18840 -23.913 <.0001

control 12 - OA 16 -0.6495 0.0293 18844 -22.190 <.0001

control 12 - control 18 -1.1126 0.0301 18839 -36.916 <.0001

control 12 - OA 18 -1.0082 0.0298 18844 -33.854 <.0001

control 12 - control 20 -1.6324 0.0296 18840 -55.217 <.0001

control 12 - OA 20 -1.2556 0.0289 18844 -43.484 <.0001

OA 12 - control 14 -0.0701 0.0297 18844 -2.365 0.3473

OA 12 - OA 14 -0.3142 0.0277 18840 -11.341 <.0001

OA 12 - control 16 -0.4552 0.0275 18843 -16.547 <.0001

OA 12 - OA 16 -0.3915 0.0261 18838 -14.972 <.0001

OA 12 - control 18 -0.8546 0.0281 18844 -30.409 <.0001

OA 12 - OA 18 -0.7501 0.0268 18839 -27.964 <.0001

OA 12 - control 20 -1.3743 0.0274 18844 -50.147 <.0001

OA 12 - OA 20 -0.9975 0.0257 18837 -38.785 <.0001

control 14 - OA 14 -0.2441 0.0307 18844 -7.949 <.0001

control 14 - control 16 -0.3850 0.0301 18841 -12.785 <.0001

control 14 - OA 16 -0.3213 0.0296 18844 -10.846 <.0001

control 14 - control 18 -0.7845 0.0304 18839 -25.768 <.0001

control 14 - OA 18 -0.6800 0.0301 18844 -22.592 <.0001

control 14 - control 20 -1.3042 0.0299 18840 -43.593 <.0001

control 14 - OA 20 -0.9274 0.0292 18844 -31.716 <.0001

OA 14 - control 16 -0.1410 0.0291 18844 -4.850 0.0001

OA 14 - OA 16 -0.0773 0.0274 18839 -2.816 0.1310

OA 14 - control 18 -0.5404 0.0296 18844 -18.254 <.0001

OA 14 - OA 18 -0.4359 0.0279 18838 -15.602 <.0001

OA 14 - control 20 -1.0601 0.0290 18844 -36.518 <.0001

OA 14 - OA 20 -0.6833 0.0271 18839 -25.196 <.0001

control 16 - OA 16 0.06370 0.0276 18844 2.311 0.3814

control 16 - control 18 -0.3994 0.0286 18838 -13.981 <.0001

control 16 - OA 18 -0.2950 0.0283 18844 -10.439 <.0001

control 16 - control 20 -0.9191 0.0279 18838 -32.972 <.0001

control 16 - OA 20 -0.5424 0.0272 18844 -19.975 <.0001

OA 16 - control 18 -0.4631 0.0282 18844 -16.445 <.0001

OA 16 - OA 18 -0.3587 0.0266 18838 -13.501 <.0001

OA 16 - control 20 -0.9829 0.0275 18844 -35.771 <.0001

OA 16 - OA 20 -0.6061 0.0256 18837 -23.701 <.0001

control 18 - OA 18 0.1045 0.0288 18844 3.623 0.0109

control 18 - control 20 -0.5197 0.0283 18838 -18.361 <.0001

control 18 - OA 20 -0.1430 0.0277 18844 -5.155 <.0001

OA 18 - control 20 -0.6242 0.0282 18844 -22.154 <.0001

OA 18 - OA 20 -0.2474 0.0262 18838 -9.428 <.0001

control 20 - OA 20 0.3768 0.0271 18844 13.923 <.0001

**Sum Ventilation**

**Comparisons Estimate SE df t-ratio *p*-value**

control 12 - OA 12 -0.0374 0.200 1608 -0.187 1.0000

control 12 - control 14 -0.1295 0.188 1604 -0.688 0.9996

control 12 - OA 14 -0.4612 0.206 1607 -2.241 0.4292

control 12 - control 16 -0.3755 0.193 1604 -1.947 0.6364

control 12 - OA 16 -0.1526 0.198 1606 -0.771 0.9989

control 12 - control 18 -0.7290 0.193 1605 -3.779 0.0063

control 12 - OA 18 -0.5101 0.194 1607 -2.629 0.2048

control 12 - control 20 -2.8814 0.187 1604 -15.416 <.0001

control 12 - OA 20 -0.9113 0.196 1607 -4.657 0.0002

OA 12 - control 14 -0.0921 0.194 1608 -0.474 1.0000

OA 12 - OA 14 -0.4238 0.201 1605 -2.103 0.5252

OA 12 - control 16 -0.3380 0.199 1608 -1.703 0.7940

OA 12 - OA 16 -0.1152 0.194 1605 -0.595 0.9999

OA 12 - control 18 -0.6916 0.201 1606 -3.436 0.0214

OA 12 - OA 18 -0.4727 0.190 1604 -2.482 0.2790

OA 12 - control 20 -2.8440 0.195 1607 -14.592 <.0001

OA 12 - OA 20 -0.8739 0.192 1604 -4.549 0.0002

control 14 - OA 14 -0.3317 0.199 1607 -1.664 0.8155

control 14 - control 16 -0.2459 0.187 1604 -1.316 0.9502

control 14 - OA 16 -0.0231 0.191 1607 -0.121 1.0000

control 14 - control 18 -0.5994 0.187 1605 -3.199 0.0455

control 14 - OA 18 -0.3805 0.187 1608 -2.034 0.5747

control 14 - control 20 -2.7519 0.181 1605 -15.173 <.0001

control 14 - OA 20 -0.7818 0.189 1608 -4.136 0.0015

OA 14 - control 16 0.0858 0.204 1608 0.421 1.0000

OA 14 - OA 16 0.3086 0.197 1604 1.571 0.8623

OA 14 - control 18 -0.2677 0.206 1604 -1.298 0.9542

OA 14 - OA 18 -0.0488 0.193 1604 -0.252 1.0000

OA 14 - control 20 -2.4202 0.200 1604 -12.086 <.0001

OA 14 - OA 20 -0.4501 0.195 1604 -2.305 0.3864

control 16 - OA 16 0.2229 0.196 1607 1.139 0.9806

control 16 - control 18 -0.3535 0.192 1605 -1.843 0.7077

control 16 - OA 18 -0.1346 0.192 1608 -0.703 0.9995

control 16 - control 20 -2.5060 0.186 1605 -13.470 <.0001

control 16 - OA 20 -0.5359 0.193 1608 -2.771 0.1473

OA 16 - control 18 -0.5764 0.198 1603 -2.908 0.1041

OA 16 - OA 18 -0.3575 0.185 1604 -1.930 0.6486

OA 16 - control 20 -2.7288 0.192 1603 -14.204 <.0001

OA 16 - OA 20 -0.7587 0.187 1604 -4.057 0.0021

control 18 - OA 18 0.2189 0.194 1604 1.128 0.9819

control 18 - control 20 -2.1525 0.185 1604 -11.614 <.0001

control 18 - OA 20 -0.1823 0.196 1604 -0.930 0.9955

OA 18 - control 20 -2.3713 0.188 1604 -12.607 <.0001

OA 18 - OA 20 -0.4012 0.184 1604 -2.184 0.4685

control 20 - OA 20 1.9701 0.190 1604 10.373 <.0001

**A. sternalis**

**Comparisons Estimate SE df t-ratio *p*-value**

control 12 - OA 12 -0.2719 0.139 1606 -1.958 0.6288

control 12 - control 14 -0.1583 0.131 1604 -1.213 0.9705

control 12 - OA 14 -0.0178 0.143 1607 -0.125 1.0000

control 12 - control 16 -0.5568 0.134 1604 -4.167 0.0013

control 12 - OA 16 -0.2239 0.137 1607 -1.633 0.8319

control 12 - control 18 -0.3773 0.134 1604 -2.822 0.1298

control 12 - OA 18 -0.5984 0.134 1607 -4.450 0.0004

control 12 - control 20 -1.0459 0.130 1604 -8.075 <.0001

control 12 - OA 20 -1.1045 0.136 1607 -8.143 <.0001

OA 12 - control 14 0.1136 0.135 1606 0.844 0.9979

OA 12 - OA 14 0.2542 0.140 1604 1.820 0.7223

OA 12 - control 16 -0.2848 0.138 1606 -2.070 0.5489

OA 12 - OA 16 0.0480 0.134 1604 0.358 1.0000

OA 12 - control 18 -0.1054 0.139 1607 -0.755 0.9991

OA 12 - OA 18 -0.3264 0.132 1604 -2.473 0.2837

OA 12 - control 20 -0.7739 0.135 1607 -5.730 <.0001

OA 12 - OA 20 -0.8326 0.133 1604 -6.254 <.0001

control 14 - OA 14 0.1406 0.138 1607 1.018 0.9912

control 14 - control 16 -0.3984 0.130 1604 -3.076 0.0655

control 14 - OA 16 -0.0656 0.133 1607 -0.495 1.0000

control 14 - control 18 -0.2190 0.130 1604 -1.687 0.8032

control 14 - OA 18 -0.4400 0.130 1607 -3.394 0.0245

control 14 - control 20 -0.8875 0.126 1604 -7.061 <.0001

control 14 - OA 20 -0.9462 0.131 1607 -7.224 <.0001

OA 14 - control 16 -0.5390 0.141 1607 -3.819 0.0054

OA 14 - OA 16 -0.2061 0.136 1604 -1.514 0.8869

OA 14 - control 18 -0.3595 0.143 1608 -2.516 0.2607

OA 14 - OA 18 -0.5806 0.134 1604 -4.330 0.0007

OA 14 - control 20 -1.0281 0.139 1607 -7.408 <.0001

OA 14 - OA 20 -1.0867 0.135 1604 -8.030 <.0001

control 16 - OA 16 0.3328 0.136 1607 2.456 0.2937

control 16 - control 18 0.1795 0.133 1604 1.350 0.9417

control 16 - OA 18 -0.0416 0.133 1607 -0.313 1.0000

control 16 - control 20 -0.4891 0.129 1604 -3.794 0.0060

control 16 - OA 20 -0.5477 0.134 1607 -4.087 0.0019

OA 16 - control 18 -0.1534 0.137 1608 -1.117 0.9831

OA 16 - OA 18 -0.3745 0.128 1604 -2.917 0.1016

OA 16 - control 20 -0.8220 0.133 1608 -6.173 <.0001

OA 16 - OA 20 -0.8806 0.130 1604 -6.794 <.0001

control 18 - OA 18 -0.2211 0.135 1607 -1.643 0.8267

control 18 - control 20 -0.6686 0.128 1604 -5.206 <.0001

control 18 - OA 20 -0.7272 0.136 1607 -5.353 <.0001

OA 18 - control 20 -0.4475 0.130 1607 -3.433 0.0216

OA 18 - OA 20 -0.5061 0.127 1604 -3.975 0.0029

control 20 - OA 20 -0.0586 0.132 1607 -0.445 1.0000

**Sum gills**

**Comparisons Estimate SE df t-ratio *p*-value**

control 12 - OA 12 -0.0872 0.0832 1606 -1.047 0.9892

control 12 - control 14 -0.0419 0.0782 1604 -0.536 0.9999

control 12 - OA 14 -0.1840 0.0855 1606 -2.152 0.4910

control 12 - control 16 -0.2961 0.0801 1604 -3.698 0.0085

control 12 - OA 16 -0.2346 0.0822 1606 -2.855 0.1193

control 12 - control 18 -0.2450 0.0801 1604 -3.058 0.0690

control 12 - OA 18 -0.3482 0.0806 1606 -4.321 0.0007

control 12 - control 20 -0.7289 0.0776 1604 -9.390 <.0001

control 12 - OA 20 -0.5927 0.0813 1606 -7.292 <.0001

OA 12 - control 14 0.0452 0.0807 1605 0.560 0.9999

OA 12 - OA 14 -0.0968 0.0837 1604 -1.157 0.9784

OA 12 - control 16 -0.2089 0.0824 1605 -2.534 0.2509

OA 12 - OA 16 -0.1475 0.0804 1604 -1.835 0.7130

OA 12 - control 18 -0.1579 0.0836 1606 -1.889 0.6770

OA 12 - OA 18 -0.2610 0.0791 1604 -3.300 0.0332

OA 12 - control 20 -0.6417 0.0809 1606 -7.927 <.0001

OA 12 - OA 20 -0.5056 0.0798 1604 -6.337 <.0001

control 14 - OA 14 -0.1420 0.0828 1606 -1.716 0.7868

control 14 - control 16 -0.2541 0.0776 1604 -3.274 0.0361

control 14 - OA 16 -0.1927 0.0794 1606 -2.426 0.3106

control 14 - control 18 -0.2031 0.0778 1604 -2.610 0.2136

control 14 - OA 18 -0.3063 0.0777 1606 -3.942 0.0034

control 14 - control 20 -0.6869 0.0753 1604 -9.120 <.0001

control 14 - OA 20 -0.5508 0.0785 1606 -7.016 <.0001

OA 14 - control 16 -0.1121 0.0846 1606 -1.326 0.9478

OA 14 - OA 16 -0.0507 0.0816 1604 -0.621 0.9998

OA 14 - control 18 -0.0611 0.0857 1606 -0.713 0.9994

OA 14 - OA 18 -0.1642 0.0803 1604 -2.044 0.5677

OA 14 - control 20 -0.5449 0.0832 1606 -6.552 <.0001

OA 14 - OA 20 -0.4087 0.0811 1604 -5.040 <.0001

control 16 - OA 16 0.0614 0.0812 1606 0.756 0.9991

control 16 - control 18 0.0511 0.0797 1604 0.641 0.9998

control 16 - OA 18 -0.0521 0.0796 1606 -0.655 0.9997

control 16 - control 20 -0.4328 0.0773 1604 -5.602 <.0001

control 16 - OA 20 -0.2966 0.0803 1606 -3.693 0.0087

OA 16 - control 18 -0.0104 0.0823 1607 -0.126 1.0000

OA 16 - OA 18 -0.1136 0.0769 1604 -1.476 0.9016

OA 16 - control 20 -0.4942 0.0798 1607 -6.194 <.0001

OA 16 - OA 20 -0.3581 0.0777 1604 -4.610 0.0002

control 18 - OA 18 -0.1032 0.0806 1606 -1.280 0.9582

control 18 - control 20 -0.4839 0.0770 1604 -6.287 <.0001

control 18 - OA 20 -0.3477 0.0814 1606 -4.271 0.0009

OA 18 - control 20 -0.3807 0.0781 1606 -4.872 0.0001

OA 18 - OA 20 -0.2445 0.0763 1604 -3.204 0.0448

control 20 - OA 20 0.1362 0.0789 1606 1.726 0.7807

**R script for linear mixed effects model**

require(lme4)

require(emmeans)

## standardization

dataset$ParameterCentered <- scale(dataset$Parameter, center = TRUE, scale= TRUE)

## random intercept models

ParameterRandIntModel <- lmer(ParameterCentered ~ Condition * Temperature + (1 | Animal), data = dataset)

## likelihood-ratio tests

# create reduced models

ParameterNullTemp <- lmer(ParameterCentered ~ Temperature + (1 | Animal), data = dataset)

ParameterNullCond <- lmer(ParameterCentered ~ Condition + (1 | Animal), data = dataset)

ParameterNullTempCond <- lmer(ParameterCentered ~ Condition + Temperature + (1 | Animal), data = dataset)

# compare models

anova(ParameterNullTemp, ParameterRandIntModel)

anova(ParameterNullCond, ParameterRandIntModel)

anova(ParameterNullTempCond, ParameterRandIntModel)

## diagnostic plots

hist(residuals(ParameterRandIntModel), main = "Residuals Parameter")

qqnorm(resid(ParameterRandIntModel))

qqline(resid(ParameterRandIntModel))

## all-pairwise Comparisons

Parametercomparisons <- emmeans(ParameterRandIntModel, specs = pairwise ~ Condition:Temperature, pbkrtest.limit = numberOfObservations)

*Note: The detailed output of the linear mixed effects model is shown in fig. S2 and tables S1-S3*
